# Are neuromuscular adaptations present in people with recurrent spinal pain during a period of remission? a systematic review

**DOI:** 10.1371/journal.pone.0249220

**Published:** 2021-04-01

**Authors:** Valter Devecchi, Alison B. Rushton, Alessio Gallina, Nicola R. Heneghan, Deborah Falla

**Affiliations:** Centre of Precision Rehabilitation for Spinal Pain (CPR Spine), School of Sport, Exercise and Rehabilitation Sciences, College of Life and Environmental Sciences, University of Birmingham, Birmingham, United Kingdom; University of Illinois at Urbana-Champaign, UNITED STATES

## Abstract

A plethora of evidence supports the existence of neuromuscular changes in people with chronic spinal pain (neck and low back pain), yet it is unclear whether neuromuscular adaptations persist for people with recurrent spinal pain when in a period of remission. This systematic review aimed to synthesise the evidence on neuromuscular adaptations in people with recurrent spinal pain during a period of remission. Electronic databases, grey literature, and key journals were searched from inception up to the 4^th^ of September 2020. Eligibility criteria included observational studies investigating muscle activity, spine kinematics, muscle properties, sensorimotor control, and neuromuscular performance in adults (≥ 18 years) with recurrent spinal pain during a period of remission. Screening, data extraction, and quality assessment (Newcastle-Ottawa Scale) were conducted independently by two reviewers. Data synthesis was conducted per outcome domain. A meta-analysis with a random-effects model was performed where possible. The overall strength of evidence was rated using the Grading of Recommendations, Assessment, Development and Evaluation guidelines (GRADE). From 8292 records, 27 and five studies were included in a qualitative and quantitative synthesis, respectively. Very low level of evidence supports muscle activity changes in people with recurrent low back pain, especially greater co-contraction, redistribution of muscle activity, and delayed postural control of deeper trunk muscles. Reduced range of motion of the lumbar spine was also found. Meaningful conclusions regarding other outcome domains or people with recurrent neck pain could not be drawn. In conclusion, people with recurrent low back pain during a period of remission show muscle activity and spine kinematics adaptations. Future research should investigate the long-term impact of these changes, as well as adaptations in people with recurrent neck pain.

## Introduction

In 2015 more than 500 million and approximately 350 million people worldwide experienced low back pain (LBP) and neck pain (NP), respectively [1, 2]. Complete remission, described as the absence of symptoms, is rare in spinal pain (LBP or NP) that is often characterised by recurrences [3]. In this regard, Stanton et al. [4] provided a definition of recurrent LBP (rLBP), that is “LBP which has occurred at least 2 times over the past year with each episode of LBP lasting at least 24hrs, with a pain intensity of >2 on an 11-point numeric rating scale, and with at least a 30 day pain-free period between episodes”.

Although the experience of previous pain episodes is a significant risk factor for new episodes of pain [5], clinical heterogeneity exists in people with spinal pain and several other factors may contribute to recurrent episodes of pain. Neuromuscular adaptations (e.g. changes in muscle activity, kinematics, muscle properties, sensorimotor control, and performance) have been extensively examined in people with spinal pain as changes in these features may contribute to pain persistence or recurrence [6–8]. For example, adaptations in muscle activity, spine kinematics, and sensorimotor control have been reported in symptomatic people [9–12] and some studies suggest that these changes extend beyond the duration of a painful episode and could lead to potential long-term consequences, such as pain recurrence [13–15]. In support of this, current theories on pain and movement suggest that the new motor strategies which are adopted in the presence of pain could lead to suboptimal loading of the spine thereby contributing to persistent or recurrent symptoms [6–8].

Although evidence on neuromuscular adaptations in people with chronic spinal pain has been extensively synthesised [16–19], there is a need to conduct the current systematic review to understand whether neuromuscular adaptations are present in people experiencing recurrent spinal pain during a period of remission. If neuromuscular adaptations are detected during a period of remission, this would indicate that nociception/pain does not have to be present for these adaptations to exist/persist. The results of this systematic review stand to identify neuromuscular features to examine in longitudinal studies with the aim of understanding whether the presence or extent of these features is predictive of pain recurrence. Translated into practice, the findings of this systematic review could provide new insight for the management of neuromuscular function in people with spinal pain, as well as promote the development of secondary prevention strategies. Therefore, the present systematic review aims to synthesise the evidence on neuromuscular adaptations in people with recurrent spinal pain (during a period of remission) when compared to a population without a history of spinal pain.

## Methods

### Protocol and registration

This systematic review was conducted according to a pre-defined published and registered protocol [20] on the International Prospective Register of Systematic Reviews (PROSPERO; CRD42019141527) on 23/07/2019. This review is reported here following the Preferred Reporting Items for Systematic Review and Meta-Analyses (PRISMA) statement (see S1 Table) [21].

### Eligibility criteria

Eligibility were defined using an adapted PICOS framework (P—population, I–Interventions, C–Comparator/Control, O–Outcomes, and S–Study design) and criteria are reported in Table 1 [20, 21].

**Table 1:** Eligibility criteria in accordance with the PICOS framework.

**POPULATION**			
Adults (age ≥ 18) with recurrent idiopathic spinal pain (two or more episodes of neck or low back pain in the past) and tested during a period of remission.			
**Exclusion criteria**: neuropathic and radiating pain, spine injury/trauma, pregnancy			
**INTERVENTIONS**			
**Interventions of interest are represented by the use of:**			
◾ Surface and intramuscular electromyography			
◾ Ultrasound			
◾ Muscle functional magnetic resonance imaging (mfMRI)			
◾ Motion analysis system, optoelectronic systems, inertial measurement unit sensors, electrogoniometer			
◾ Ultrasound			
◾ MRI / mfMRI			
◾ Dynamometry			
◾ Performance tests			
**COMPARATOR / CONTROL**			
People without a history of spinal pain as control group			
**OUTCOMES OF INTEREST**			
**Concept of interest**	**Broad Outcome Domains**	**Narrow Outcome Domains**	**Outcome measures**
	Muscle activity	◾ Amplitude and its variability	• Average rectified value
		◾ Timing and its variability	• Root mean square
			• Onset of activity
			• Change of muscle
			• thickness
			• Transverse relaxation time
Neuromuscular adaptations	Spine kinematics	◾ Active range of motion	Based on the task and equipment used (e.g. residuals, Jerk)
(spine region)		◾ Motor variability	
		◾ Quality of movement	
	Sensorimotor control	◾ Proprioception	
			• Joint reposition error
	Muscle properties	◾ Total cross-sectional area (CSA)	• Muscle thickness
		◾ Muscle CSA	• Transverse relaxation time
		◾ Fatty infiltration	
	Neuromuscular performance	◾ Strength	• Average/Peak force
		◾ Endurance/fatigue	• Time to task failure
			• Borg scale
			• EMG features (frequency)
**STUDY DESIGN**			
Observational studies represented the design of interest as suggested by a preliminary scoping search			

### Information sources and search strategy

The search was conducted from inception up to 4^th^ September 2020 by one reviewer (VD). Databases searched were MEDLINE (OVID interface), EMBASE (OVID interface), CINAHL (EBSCO interface), ZETOC, Google Scholar, PubMed, and Web of Science. Reference lists of included studies and relevant reviews were checked. Moreover, hand searching was conducted for relevant journals (Journal of Orthopaedic and Sports Physical Therapy, Clinical Biomechanics, The Clinical Journal of Pain, Spine, Musculoskeletal Science and Practice, and the Journal of Electromyography and Kinesiology).

The search strategy was developed from the PICOS framework (Table 1) and medical subject headings (MESH) were used where appropriate. The search strategy used in MEDLINE (OVID interface) is reported in S1 File. For other databases, the search strategy was adapted ensuring consistency. The British National Bibliography, OpenGrey, and dissertation abstracts were searched to screen grey literature and reduce the risk of publication bias [22].

### Study selection

Records were retrieved from databases and duplicates removed by one reviewer (VD). Using a piloted electronic screening tool developed using the eligibility criteria reported in Table 1, two reviewers (VD, AG) conducted title and abstract screening. Then, potentially relevant full-text records were independently screened by the two reviewers. During both screenings, disagreement after discussion was resolved by a third reviewer (DF), or the study’s author was contacted for additional information. When reviewers contacted authors, an initial email was sent asking for study information; when a reply was not obtained after fifteen days, a second email was sent. When eligibility information from relevant studies was not received, studies were excluded, and reasons are reported in S2 Table. If multiple records of the same study were identified, they were collated [21, 22]. The kappa statistic was used to assess agreement between the two reviewers [21].

### Data collection process and data items

Data extraction was conducted independently by two reviewers (VD, AG) using a customised data extraction sheet. When more than three groups were present in a study, data were extracted for the comparison between the control and recurrent spinal pain groups. When text and tables were not sufficient to obtain study results, data were extracted from figures using the WebPlotDigitizer software in accordance with Higgins et al [22]. Missing data were retrieved by contacting authors on two occasions as described above and where a reply was not obtained, the data were considered irretrievable. However, the study was retained using the available information.

### Quality assessment

Methodological quality of the included studies was assessed independently by two reviewers (VD, AG) using the Newcastle-Ottawa Scale (NOS) for case-control studies [23]. Disagreement was resolved through discussion. As there is no consensus on the optimal study quality or risk of bias tools for observational studies [24], the NOS was chosen because it is validated, adaptable, and quick to complete [25, 26]. In the NOS, participant characteristics and outcomes are assessed in three dimensions; selection, comparability, exposure, and for each study a star rating is designated (from 0 to a maximum of 9) [23]. Overall, three categories were identified; 0–3 = poor quality, 4–7 = fair quality, or 8–9 = good quality [20].

### Summary measures and synthesis of results

Results were summarised per outcome domain and reported in a table of main findings. Binary variable results were reported using the risk ratio, and for continuous variables, using mean and standard deviation (SD). Where different values were reported (such as standard error or confidence interval), SD was calculated [22]. Differences between the control and recurrent spinal pain groups were summarised using the standardised mean difference (SMD) and 95% confidence intervals (95% CI).

Quantitative synthesis using a random-effect meta-analysis was conducted when consistency across studies was met [27]. Clinical and methodological heterogeneity across studies was explored by the two reviewers considering spine region, task performed and outcome measure reported for each outcome domain. Where consistencies across studies were observed, statistical heterogeneity was analysed using the *I*^*2*^ statistic with an a priori cut-off defining substantial heterogeneity (*I*^*2*^ > 50%) [20, 22]. When statistical heterogeneity was found, possible reasons were investigated through subgroup analyses [22] and results were reported narratively. All analyses were computed in RevMan software (v.5.3 Cochrane Collaboration) [28]. The results from outcome domains were grouped and described narratively [29].

### Quality of evidence

Quality of evidence was assessed per outcome domain using the Grading of Recommendations Assessment, Development, and Evaluation (GRADE) approach [30]. Initially, low quality of evidence was assigned to each outcome domain since only observational studies were included [31]. Then, the quality of evidence was rated considering five factors (limitations, inconsistency, indirectness, imprecision, publication bias) and summarised in a table [31]. The NOS score for individual studies was integrated into the GRADE approach to define the study limitations of evidence (for each outcome domain) [32]. Therefore, when evidence was mainly obtained from poor methodological studies (NOS ≤ 3), limitations were described as serious. No limitation was reported with fair methodological studies (4 ≤ NOS ≤ 7). Finally, quality of evidence was upgraded when good methodological studies accounted for most of the findings (NOS ≥ 8) [32]. Overall, level of evidence was identified as ‘High’, ‘Moderate’, ‘Low’, or ‘Very Low’ [31].

### Additional analyses

When possible, two subgroup analyses were conducted based on the definition of recurrent LBP provided by Stanton et al [8]. Firstly, considering people during a period of remission with no pain at all versus those with minimal pain (VAS > 0), and secondly based on the number of painful episodes during the previous year (≥ 2 versus < 2 or not reported). This approach was adopted to avoid the exclusion of relevant studies.

## Results

### Flow of studies

**Fig 1:**
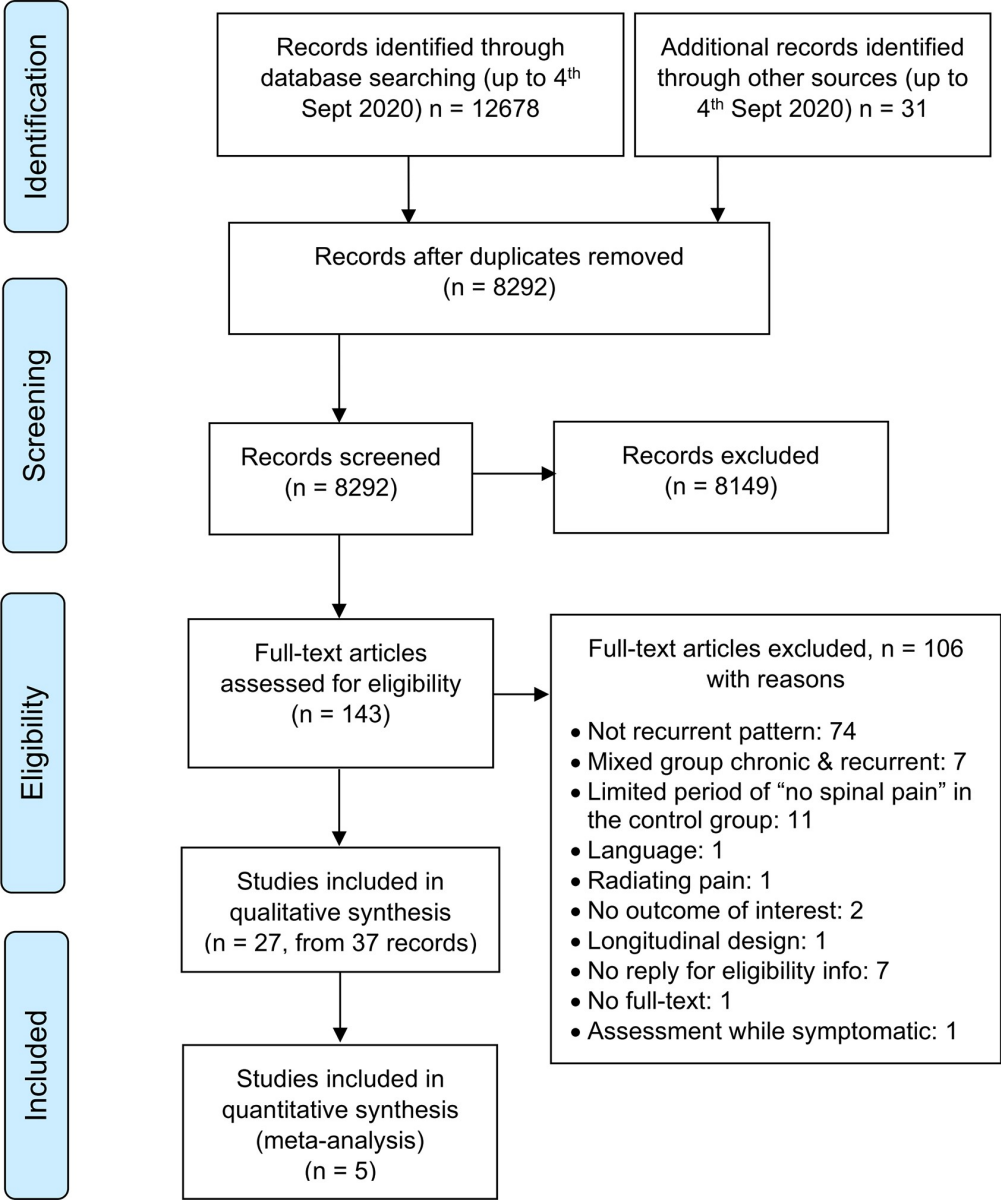
PRISMA flow diagram of search and selection of studies.

The database search retrieved 11850 records and the hand-searching an additional 31 records (Fig 1). After removal of duplicates, 8292 records were screened by title and abstract by the two reviewers with an agreement of *K* = 0.76. Full-text screening was conducted on 143 articles and the agreement between reviewers was *K* = 0.88. Ten authors were contacted, and replies were obtained from six of them (see S2 File).

From the 37 records included, multiple records of the same study were identified and collated (see S3 File). Finally, 27 studies were obtained for qualitative synthesis, and five of them were also suitable for quantitative synthesis. Excluded studies are reported in the table ‘Characteristic of excluded studies’ and specific reasons are provided (see S2 Table).

### Characteristics of included studies

Of the 27 included studies, one investigated people with recurrent NP (rNP) [33] and all others investigated recurrent LBP (rLBP). The 27 included studies reflected 30 people with rNP and 500 with rLBP (mean age ranged 21.5–46.5 years). Only six studies adopted the complete definition of “recurrent pain” including people with two or more painful episodes over the previous year [13, 34–39]. Participants were assessed during a pain remission period in 15 studies, whereas in the other 12 studies, participants reported minimal pain (mean level between 0.12 and 3.5 on a visual or numerical rating scale). Characteristics of included studies are described in detail in Table 2.

**Table 2:** Characteristics of the included studies.

Study	Control group	Recurrent spinal pain group	Outcome domain	Task	Measurement tool and body region investigated
Applegate et al., 2019 [40] Applegate et al., 2018 [41]	n = 24 12 (50%) male Age 29.2±10.8 y BMI 24.8 ± 3.43 kg/m^2^ W 73.3±12.7 kg H 1.70 ± 0.05 m	n = 24 (rLBP) 12 (50%) male Age 24.3±7.3 y; BMI 24.2 ± 3.43 kg/m^2^ W 71.4±12.7 kg; H 1.70 ± 0.05 m *N° of recurrent episodes*: > 1 episode of LBP *Pain during the assessment*: NRS: 1.4±0.4	Neuromuscular performance: • Endurance • Strength	Sørensen test	Surface EMG: • ES (L2-L4) Dynamometry: • Back extensors
Claus et al., 2018 [42]	n = 14 14 (100%) male Age 22 ± 8 y W 71 ± 10 kg H 1.78 ± 0.08 m	n = 10 (rLBP) 10 (100%) male Age 25 ± 5 y W 74 ± 10 kg; H 1.78 ± 0.06 m *N° of recurrent episodes*: > 1 episode of LBP in the past 2 years *Pain during the assessment*: Pain-free	Muscle activity: • Amplitude	Sitting in different postures	3D electromagnetic system: • Thoracic/lumbar spine (T1, T5, T10, L3, S2) Intramuscular EMG: • LT (T11), IL (T11 / L2), dMF and sMF (L4), TrA Surface EMG: • OE, OI, RA
Crosbie et al., 2013 [43]	n = 20 7 (35%) male Age 28.6±5.4 y BMI 23.0±2.4 kg/m^2^ W 67±11 kg H 170±9 m	n = 20 (rLBP) 8 (40%) male Age 34.0±13.3 y; BMI 24.5±3.6 kg/m^2^ W 72±15 kg; H 170±12 m *N° of recurrent episodes*: ≥ 2 episodes (range 3–25) *Pain during the assessment*: VAS: 1.8 (range 0–2.4)	Spine Kinematics: • Range of motion • Timing	Reaching task	3D electromagnetic tracking system: • T1 / T6 / L1 / S2
D’hooge et al., 2013 [44]	n = 14 6 (43%) male Age 25±6 y BMI 22.0 ± 2.9 kg/m^2^ W 61 ± 12 kg H 1.67 ± 0.11 m	n = 11 (rLBP) 7 (64%) male Age 25±6 y; BMI 24.5 ± 2.5 kg/m^2^ W 78 ± 16 kg; H 1.77 ± 0.11 m *N° of recurrent episodes*: ≥ 2 episodes *Pain during the assessment*: Symptom remission	Muscle activity: • Amplitude	Rapid voluntary trunk flexion	Intramuscular EMG: • dMF (L4) • sMF (L4) Surface EMG: • Lumbar ES (L4) • Thoracic ES (T9) • Latissimus dorsi (T9) • OE, OI, RA
D’hooge et al., 2012 [45] D’Hooge et al., 2013 [46]	n = 13 6 (46%) male Age 32.1±10.6 y W 74.9±13.3 kg H 1.76±0.09 m	n = 13 (rLBP) 6 (46%) male Age 32.1±11.5 y W 74.6±15.3 kg; H 1.78±0.09 m *N° of recurrent episodes*: ≥ 2 episodes *Pain during the assessment*: Pain-free	Muscle properties: • Tissue characteristics Muscle activity: • Recruitment	Rest and a low-load trunk extension exercise	mfMRI (L4): • MF • ES • Psoas • Quadratus lumborum
Elsig et al., 2014 [33]	n = 30 0 (0%) male Age 37.2 ±13.5 y W Not reported H Not reported	n = 30 (rNP) 0 (0%) male Age 36.9 ±13.6 y; W Not reported; H Not reported *N° of recurrent episodes*: > 1 episode of neck pain *Pain during the assessment*: VAS: 3.13±2.01	Sensorimotor control: • Proprioception	Cervicocephalic relocation test	Pressure Biofeedback: • Deep neck flexors
			Neuromuscular performance: • Strength	Craniocervical Flexion Test	
Fenety and Kumar, 1992 [47]	n = 12 0 (0%) male Age 20.8 ± 2.4 y W 58.9 ± 3.5 kg H 1.62 ± 0.05 m	n = 10 (rLBP) 0 (0%) male Age 21.5 ± 1.9 y; W 63.8 ± 6.3 kg; H 1.67 ± 0.04 m *N° of recurrent episodes*: ≥ 2 episodes in the preceding 2 year *Pain during the assessment*: No pain during tests	Spine kinematics: • Active ROM	Spine full flexion and full extension	Sagittal plane photographs of the spine: • Angle L1 / sacrum (S2)
			Neuromuscular performance: • Strength	Concentric and eccentric trunk flexion/extension	Isokinetic dynamometer: • Trunk flexors / extensors
Ferreira et al., 2004 [48]	n = 10 Age 32.7 ± 10.6 y W 68.2 ± 12.6 kg H 1.60 ± 0.38 m	n = 10 (rLBP) Age 27.8 ± 5.1 y; W 68.6 ± 13.1 kg; H 1.72 ± 0.1m *N° of recurrent episodes*: ≥ 2 episodes in the preceding 2 year *Pain during the assessment*: No pain during tests	Muscle activity: • Recruitment • Amplitude	Knee flexion and extension in supine position	Ultrasound imaging: • TrA, OI, OE Intramuscular EMG: • TrA, OI, OE
Gorbet et al., 2010 [49]	n = 30 Not reported Age 21.4 ± 0.6 y W 74.45 ± 2.71 kg H 1.75 ± 0.02 m	n = 30 (rLBP) Not reported Age 24.5 ± 1.6 y W 79.4 ± 3.5 kg; H 1.76 ± 0.02 *N° of recurrent episodes*: ≥ 3 episodes previous year or ≥ 5 lifetime *Pain during the assessment*: Pain remission	Muscle activity: • Recruitment	Abdominal Drawing-In maneuver: • Supine • Quadruped exercise	Ultrasound imaging: • TrA
Grimstone and Hodges, 2003 [50]	n = 10 Not reported Age 26±5.4 y W 66±15.1 kg H 1.71±0.10 m	n = 10 (rLBP) Not reported Age 32±8.3 y; W 69±14.7 kg; H 1.73±0.10 m *N° of recurrent episodes*: LBP of at least 18 months’ duration + at least one episode of pain per year *Pain during the assessment*: Little (< 2 VAS) or No pain	Spine Kinematics: • Trunk movement	Standing with three breathing conditions: • quiet breathing • hypercapnoea • increased tidal volume	Six movement sensors: • L2 / L5, pelvis anterior and posterior
Himes et al., 2012 [38]	n = 24 2 (8%) male Age 26±5 y W 68.0±9.3 kg H 169.7±8.2 m	n = 23 (rLBP) 8 (35%) male Age 24±5 y; W 71.6±12.8 kg; H 171.1±0.6 m *N° of recurrent episodes*: ≥ 3 episodes in the previous year or ≥ 5 episodes over the lifetime *Pain during the assessment*: Pain free	Muscle activity: • Recruitment	Rest and side-bridge exercises	Ultrasound imaging: • TrA (right side)
Hodges and Richardson, 1996[51] Hodges and Richardson, 1998[52]	n = 15 8 (54%) male Age 29±9 y W 67±11 kg H 1.73±0.11 m	n = 15 (rLBP) 8 (54%) male; Age 30±8 y W 74±12 kg; H 1.74±0.03 m *N° of recurrent episodes*: LBP of at least 18 months’ duration + at least one episode of pain per year *Pain during the assessment*: Minimal or absent	Muscle activity: • Timing	Standing: hip flexion, extension and abduction	Intramuscular EMG: • TrA, OI, OE Surface EMG: • RA • ES (L4/L5) •Gluteus maximus, tensor fasciae latae, rectus femoris
Hodges and Richardson., 1999 [53, 54]	n = 14 7 (50%) male Age 29 ± 7.5 y W 66 ± 11 kg H 1.72 ± 0.04 m	n = 14 (rLBP) 7 (50%) male Age 30 ± 7.5 y W 63 ± 8 kg; H 1.74±0.07 m *N° of recurrent episodes*: LBP of at least 18 months’ duration + at least one episode of pain per year *Pain during the assessment*: Pain-free	Muscle activity: • Timing	Rapid arm flexion and extension	Intramuscular EMG: • TrA, OI, OE Surface EMG: • Anterior deltoid (right) • RA (left) • ES (left—L4)
Janssens et al., 2013 [39]	n = 10 Not reported Age 24±4 y BMI 20±2 kg/m^2^ W 61±12 kg H 1.72±0.08 m	n = 10 (rLBP) Not reported Age 24±3 y; BMI 21±2 kg/m^2^ W 63±8 kg; H 1.72±0.07 m *N° of recurrent episodes*: ≥ 3 episodes in the previous 6 months *Pain during the assessment*: VAS: 1.6±1.8	Neuromuscular performance: • Strength • Endurance	Bilateral anterior magnetic phrenic nerve stimulation	Esophageal and abdominal pressures transducer: • Diaphragm
Johanson et al., 2011 [55]	n = 16 5 (31%) male Age 22.7±1.7 y W 66.8±12.5 kg H 1.75±0.10 m	n = 16 (rLBP) 5 (40%) male; Age 22.0±1.1 y; W 65.5±9.6 kg; H 1.72 ± 0.11 m *N° of recurrent episodes*: ≥ 3 episodes *Pain during the assessment*: VAS: 1.6±1.8	Neuromuscular performance: • Endurance	Modified Biering-Sørensen test	Surface EMG: • IL lumborum (L2) • MF (L5)
Larsen et al., 2018 [35]	n = 26 10 (38%) male Age 23.6±4.4 y BMI 23.8±2.5 kg/m^2^	n = 27 (rLBP) 15 (56%) male Age 27.4±9.9; BMI 21.9±3.2 kg/m^2^	Muscle activity: • Amplitude	3 sessions of: • 10 steps up • 10 steps down	Surface EMG (one side): • RA, OE, and OI • IL (L2) • Longissimus (L1) • MF (L4) • Gluteus maximus • Gluteus medius
		*N° of recurrent episodes*: ≥ 2 episodes in the previous year *Pain during the assessment*: Pain-free			
MacDonald et al., 2009 [13]	n = 19 9 (47%) male Age 26±5 y W 67±11 kg H 1.73±0.09 m	n = 15 (rLBP) 7 (47%) male; Age 27±7 y; W 71±14 kg; H 1.72±0.08 m *N° of recurrent episodes*: > 2 episodes *Pain during the assessment*: Pain free	Muscle activity: • Timing	Rapid arm flexion / extension	Intramuscular EMG: • Short and long MF fibres Surface EMG: • Deltoid
MacDonald et al., 2010 [56]	n = 14 8 (57%) male Age 26±5 y W 68±12 kg H 1.74±0.10 m	n = 13 (rLBP) 6 (46%) male Age 29±7 y; W 71±14 kg; H 1.71±0.09 m *N° of recurrent episodes*: > 2 episodes *Pain during the assessment*: Period of remission	Muscle activity: • Amplitude	Predictable and unpredictable trunk loading	Intramuscular EMG: • dMF and sMF (L5)
MacDonald et al., 2011 [57]	n = 10 6 (60%) male Age 24±3 y W 62±13 kg H 1.70 ± 0.08 m	n = 8 (rLBP) 2 (25%) male Age 23±4 y; W 65±9 kg; H 1.71 ± 0.06 m *N° of recurrent episodes*: ≥ 2 episodes in the previous year *Pain during the assessment*: Pain-free	Muscle activity: • Recruitment	Active straight leg raise, crook-lying active leg raise, prone straight leg raise	Ultrasound imaging: • MF (L4-L5 and L5-S1)
Nagar et al., 2014 [58]	n = 18 12 (67%) male Age 22.7±1.7 y BMI 22.8 ± 1.91 kg/m^2^ W 69.5 ± 9.1 kg H 172.0 ± 7.7 m	n = 18 (rLBP) 5 (45%) male Age 22.0±1.1 y; BMI 22.9 ± 2.12 kg/m^2^ W 68.5 ± 7.6 kg; H 173.8 ± 6.5 m *N° of recurrent episodes*: LBP of at least 18 months’ duration + at least one episode of pain per year *Pain during the assessment*: Pain remission	Muscle properties: • CSA Muscle activity: • Recruitment	Loaded forward reach activity with and without TrA contraction	Ultrasound imaging: • OE, OI, TrA
Park et al., 2013 [59]	n = 12 9 (75%) male Age 24±2 y W 65±12 kg H 169±5 m	n = 10 (rLBP) 6 (60%) male Age 23±4 y; W 67±12 kg; H 171±11 m *N° of recurrent episodes*: > 2 episodes *Pain during the assessment*: Pain-free	Muscle activity: • Amplitude	Trunk loading task, different directions	Intramuscular EMG: • PM-t, PM-v, QL-a, QL-p Surface EMG: • Right ES (L4), right OE and OI/TrA
Park et al., 2013 [60]	n = 9 7 (78%) male Age 23±3 y W 62±8 kg H 169±5 m	n = 10 (rLBP) 6 (60%) male Age 23±4 y; W 67±12kg; H 171±11 m *N° of recurrent episodes*: > 2 episodes *Pain during the assessment*: Pain-free	Muscle activity: • Amplitude	3 sitting postures: flat, slump, short lordotic	Intramuscular EMG: • PM-t, PM-v, QL-a, and QL-p L3/L4 Surface EMG: • OE, OI/TrA, ES 3D motion analysis system: • Thoracic and lumbar spine (T1, T5, T10, L3, S2)
Phillips, 2013 [61]	n = 40 13 (33%) male Age 41.8±9.1 y W 67.4±13.2 kg H 1.71±0.1 m	n = 61 (rLBP) 27 (44%) male Age 44.1±9.8 y; W 74.9±14.2kg; H 1.72±0.1 m *N° of recurrent episodes*: ≥ 2 episodes *Pain during the assessment*: VAS: 11.5 ± 13.5	Sensorimotor control: • Proprioception	Position awareness test (end-range)	Flexible M180B electrogoniometer: • S1-L1 spinous process
Phillips, 2013 [62]	n = 50 16 (33%) male Age 43.6±11.0 y W 72.8±14.2 kg H 1.70±0.08 m	n = 50 (rLBP) 20 (44%) male Age 46.5±10.9 y; W 78.9±17.1 kg; H 1.70±0.1 m *N° of recurrent episodes*: ≥ 2 episodes *Pain during the assessment*: VAS: 35.1 ± 17.8	Sensorimotor control: • Proprioception Spine kinematics: • Active range of motion during sitting (ext-flex)	Position awareness test	Flexible M180B electrogoniometer: • S1-L1 spinous process
				From slump sitting to max extension of the low back	
Smith et al., 2016 [37] Smith et al., 2017 [63] Smith et al., 2016 [64]	n = 14 6 (43%) male Age 24.5±1.8 y W 66.7 ± 15.0 kg H 1.73 ± 0.05 m	n = 14 (rLBP) 6 (43%) male Age 26.5 ± 4.8 y W 66.7 ± 15.0 kg; H 1.73 ± 0.05 m *N° of recurrent episodes*: ≥ 2 episodes in the preceding year *Pain during the assessment*: VAS: 0.12 ± 0.24	Muscle activity: • Amplitude • Timing Spine Kinematics: ROM • Coordination	Turning while walking	Intramuscular EMG: • dMF (L4) • lumbar longissimus (L4) • thoracic longissimus (T10) Digital motion capture system: • Thorax and Pelvis
Suehiro et al., 2018 [65]	n = 20 12 (60%) male Age 27.1±7.6 y W 58.6 ± 9.2 kg H 166.1 ± 9.0 m	n = 25 (rLBP) 15 (60%) male Age 26.8 ± 5.2 y W 60.5 ± 13.4 kg; H 166.8 ± 7.9 m *N° of recurrent episodes*: ≥ 2 episodes *Pain during the assessment*: Remission period	Muscle activity: • Amplitude • Timing	Lifting task	Surface EMG: • OE, TrA/OI, ES (L1), MF(L5), ant deltoid
Sutherlin et al., 2019 [66]	n = 24 6 (25%) male Age 23±8 y W 69.8 ± 13.8 kg H 169.0 ± 8.5 m	n = 21 (rLBP) 6 (29%) male Age 25 ± 9 y; W 70.2 ± 11.8 kg; H 170.0 ± 8.0 m *N° of recurrent episodes*: > 2 episodes *Pain during the assessment*: VAS: 9 ± 13	Spine kinematics: • Joint stiffness	Landing task	3D electromagnetic motion capture system + 8 electromagnetic sensors: • C7/T1, T12/L1, L5/S1
Sutherlin et al., 2018 [67]	n = 34 10 (29%) male Age 22±7 y W 68.3 ± 13.3 kg H 169.0 ± 9.2 m BMI 23.7 ± 2.7 kg/m^2^	n = 25 (rLBP) 9(36%) male Age 25 ± 10 y; BMI 24.0 ± 3.2 kg/m^2^ W 70.2 ± 11.1 kg; H 171.2 ± 8 m *N° of recurrent episodes*: > 2 episodes *Pain during the assessment*: VAS: 15 ± 14	Muscle activity: • Recruitment	Drawing-in. Different postures: prone/supine, sitting, standing, walking	Ultrasound imaging: • Lumbar MF • TrA
Viggiani et al., 2020 [34]	n = 11 5 (46%) male Age 25.2±5.2 y W 67.4±13.3 kg H 1.71±0.10 m BMI 22.9±3.0 kg/m^2^	n = 11 4 (36%) male Age 35.8±10.9 y W 63.5±7.0 kg; H 1.72±0.06 m BMI 21.8±1.6 kg/m^2^ *N° of recurrent episodes*: ≥ 2 episodes in the previous year *Pain during the assessment*: VAS: 2.7±3.1	Muscle activity: • Amplitude	Trunk extension while standing	Surface EMG (bilaterally): • ES (T9 and L3) • Gluteus Maximus • Biceps Femoris (Long head) • OE and OI
			Spine Kinematics: • range of motion • smoothness		3D motion capture system: Trunk/pelvis angle

BMI, body mass index; CSA, cross-sectional area; dMF, deep multifidus fibres; EMG, electromyography; ES, erector spinae; H, height; IL, iliocostalis; LT, longissimus thoracic; MF, multifidus; mfMRI, muscular functional magnetic resonance imaging; NRS, numeric rating scale; OE, external oblique; OI, internal oblique; PM-t, psoas major transverse process; PM-v, psoas major vertebral body; QL-a, quadratus lumborum anterior; QL-p, quadratus lumborum posterior; RA, rectus abdominis; rLBP, recurrent low back pain; rNP, recurrent neck pain; sMF, superficial multifidus fibres; TrA, Transversus abdominis; VAS, visual analog scale; W, weight.

### Quality assessment of included studies

Methodological quality differs considerably across studies and overall scores range between poor (★★) and fair (★★★★★★) (Table 3). Poor methodology in the selection of cases was present in all studies owing to faulty definition and representativeness of people with spinal pain. Around 20% of included studies matched cases and controls for at least one factor; therefore, comparability was affected in 80% of studies.

**Table 3:** Quality evaluation of included studies.

STUDY	SELECTION^a^	COMPARABILITY^a^	EXPOSURE^a^	OVERALL
Applegate et al. [40, 41]	☆☆☆★	★★	☆★☆	4
Claus et al. [42]	☆☆☆★	☆☆	☆★☆	2
Crosbie et al. [43]	☆☆☆★	☆☆	☆★☆	2
D’Hooge et al. [44]	☆☆☆★	☆☆	☆★☆	2
D’Hooge et al. [45, 46]	☆☆☆★	☆☆	☆★☆	2
Elsig et al. [33]	☆☆☆★	★★	☆★☆	4
Fenety and Kumar [47]	☆★★★	☆★	★★☆	6
Ferreira et al. [48]	☆☆☆★	☆☆	☆★☆	2
Gorbert et al. [49]	☆☆★★	☆☆	☆★☆	3
Grimstone and Hodges [50]	☆☆☆★	☆☆	☆★☆	2
Himes et al. [38]	☆☆★★	☆☆	☆★☆	3
Hodges [51–54]	☆☆☆★	★★	☆★☆	4
Janssens et al. [39]	☆☆☆★	☆☆	☆★☆	2
Johanson et al. [55]	☆☆☆★	☆☆	★★☆	3
Larsen et al. [35]	☆☆★★	☆☆	☆★☆	3
MacDonald et al. [13]	☆☆☆★	☆☆	☆★☆	2
MacDonald et al. [56]	☆☆☆★	☆☆	☆★☆	2
MacDonald et al. [57]	☆☆☆★	☆☆	☆★☆	2
Nagar et al. [58]	☆☆★★	☆☆	☆★☆	3
Park et al. [59, 60]	☆☆☆★	☆☆	☆★☆	2
Phillips [61]	☆☆★★	☆☆	☆★☆	3
Phillips [62]	☆☆☆★	☆☆	☆★☆	2
Smith and Kulig [37, 63, 64]	☆☆★★	★★	☆★☆	5
Suehiro et al. [65]	☆☆☆★	☆☆	☆★☆	2
Sutherlin et al. [66]	☆☆★★	☆☆	☆★★	4
Sutherlin et al. [67]	☆☆★★	☆☆	☆★★	4
Viggiani et al. [34]	☆☆☆★	☆☆	☆★☆	2

^a^Each star position corresponds to the specific item evaluated in the Newcastle-Ottawa Scale (case-control)

### Results of individual studies

The main findings of each study are summarised in S3 Table. From the results retrieved in individual studies, evidence of neuromuscular adaptations in people with rLBP were found for the following outcome domains: muscle activation amplitude [34, 35, 37, 38, 42, 44, 48, 49, 56, 57, 58–60, 65, 67] and timing [13, 37, 51–54, 65], spine kinematics [34, 43, 47, 50, 62–64, 66], sensorimotor control [61, 62], muscle properties [45, 46, 58, 68], and neuromuscular performance [39–41, 46, 47, 55, 69]. For people with rNP, sensorimotor control and neck muscle performance were assessed in the single study included [33].

### Synthesis of results and additional analysis

The main findings from individual studies were grouped per outcome domain and the obtained evidence was narratively described across studies. The quality of evidence per outcome domain was summarised in accordance with GRADE and is reported in Table 4. The variability in task, target muscle and outcome measurement tool resulted in high clinical and methodological heterogeneity across studies, precluding quantitative synthesis for most of the outcome domains considered. When clinical and methodological consistency was observed, quantitative synthesis was influenced by a high statistical heterogeneity across studies (*I*^*2*^ > 50%).

**Table 4:** Quality assessment of evidence for neuromuscular changes in people with recurrent spinal pain (GRADE).

Quality assessment per outcome domain–Observational studies						
N° of patients (studies)	Limitations	Inconsistency	Indirectness	Imprecision	Publication bias	Overall
Outcome: Muscle activity–Amplitude						
235 (14)	Serious^a^	Serious^b^	Not serious	Serious^c^	Suspected^d^	Very low
Quantitative synthesis: 106 (5)	Serious^a^	Serious^b^	Not serious	Serious^c^	Suspected^d^	Very low
Outcome: Muscle activity–Timing						
69 (4)	Not serious	Not serious	Not serious	Serious^c^	Suspected^d^	Very low
Outcome: Spine kinematics						
136 (7)	Serious^a^	Serious^b^	Not serious	Serious^c^	Suspected^d^	Very low
Outcome: Sensorimotor control–Proprioception						
141 (3)	Serious^a^	Serious^b^	Not serious	Serious^c^	Suspected^d^	Very low
Outcome: Muscle properties–Tissue characteristics						
31 (2)	Serious^a^	Serious^b^	Not serious	Serious^c^	Suspected^d^	Very low
Outcome: Neuromuscular performance–Strength & Endurance						
103 (6)	Not serious	Serious^b^	Not serious	Serious^c^	Suspected^d^	Very low

Abbreviation: GRADE, Grading of Recommendations, Assessment, Development and Evaluation guidelines

^a^Poor methodological quality of included studies

^b^High level of heterogeneity

^c^Studies with moderate Confidence Intervals and small sample size

^d^Limited number of observational studies (small sample size).

#### Muscle activity

Muscle activity in people with rLBP was investigated in twelve studies (*n* = 196) of poor quality and two of fair quality (*n* = 39). No studies assessed neck muscle activity in people with rNP.

Overall, very low level evidence (Table 4) supports that people with rLBP present with greater co-contraction of abdominal and back muscles, as well as redistributed activity between lumbar extensor muscles. However, when recruitment was considered for individual muscles and compared between groups, findings were inconsistent. Evidence regarding deep trunk muscle recruitment was inconclusive.

Trunk muscle activity alterations during functional tasks (sitting, walking, step up, and step down) were described in four studies [35, 37, 42, 60]. Claus et al. [42] reported an increased activity of the longissimus and iliocostalis (SMD, 2.27; 95% CI: 1.24, 3.31 and SMD, 1.16; 95% CI: 0.29, 2.04, respectively) in people with rLBP, as well as an impaired ability to modulate the activity of the multifidus (MF) across different sitting postures. Park’s [60] results support these data showing a redistribution of muscle activity in people with rLBP; lower erector spinae activity was compensated by increased activation of other back muscles (psoas and quadratus lumborum) with a bias toward extension [60]. Nevertheless, differences in the activity of the MF were not observed by Smith and Kulig [37], when participants performed a turning task; MF activity increased between self-selected and fast speed walking but without differences across groups. An overall increase in the activity of both flexor and extensor trunk muscles was reported by Larsen et al. [35] during ten consecutive repetitions of a step task (ascent and descent).

Similar findings of trunk muscle activity when acting as prime movers or with a stabilisation role were found as well [44, 59, 65]. Overall, greater co-contraction of superficial abdominal and back muscles was found [44, 59, 65]. Through the assessment of trunk movements, D’hooge et al. [44] found a greater co-contraction index of flexor and extensor trunk muscles in people with rLBP. Similar findings were obtained by Suheiro et al. [65] investigating a lifting task. However, another study showed that the increase of paraspinal muscle activity was not homogenous across participants [59]. In accordance with a previous study investigating muscle activity while sitting, a redistribution of activity across back extensor muscles was reported during a trunk loading task [59]. Different results were reported by Viggiani et al [34]. During a trunk extension task while standing, people with rLBP showed a lower activation of the erector spinae assessed at T9 and L3 (bilaterally) [34].

Ultrasound (US) imaging was used to investigate the recruitment of abdominal muscles (transversus abdominis [TrA], internal [OI] and external oblique [OE]) and MF in five and two studies, respectively [38, 48, 49, 57, 58, 67]. The OE and OI were assessed in one study and no between-group differences were found [48].

One study found a greater thickness change of the MF during the prone straight leg raise in people with rLBP [57]. However, Sutherlin et al. [67] reported no differences between groups when participants adopted different postures or during walking.

**Fig 2:**
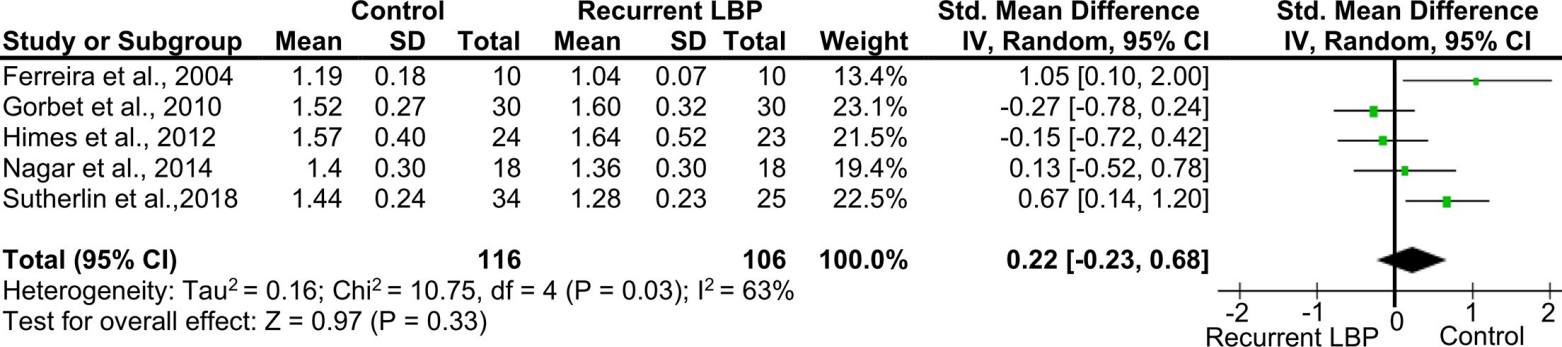
Quantitative synthesis for TrA thickness change. Studies comparing the change of TrA thickness measured with ultrasound in people with recurrent LBP and a control group. Means and standard deviations (SD) of the TrA activation ratio are reported.

Results for the recruitment of the TrA during low intensity contractions have been grouped for quantitative synthesis and are reported in Fig 2. Included studies were affected by a poor quality. The analysis was performed using a random-effects model. To account for the high statistical heterogeneity (*I*^*2*^ > 50%, Fig 2), subgroup analyses defined *a priori* were performed, but the statistical heterogeneity remained high (*I*^*2*^ > 50%).

**Fig 3:**
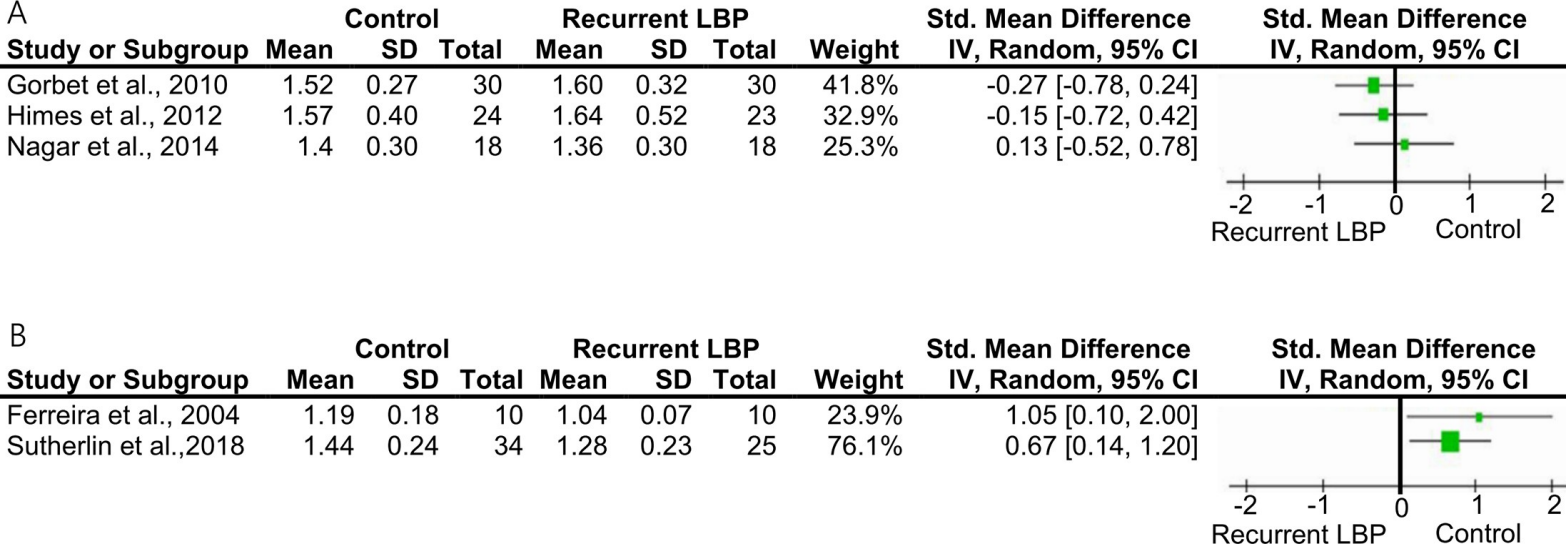
Post-hoc subgroup analysis for TrA thickness change. Forest plots of studies comparing the TrA thickness change measured with ultrasound in people with recurrent LBP and a control group. (A) Studies where participants received training and feedback before testing. (B) No practice was allowed before testing. Mean and standard deviations (SD) of the TrA activation ratio are reported.

Based on the theoretical rationale of the outcome domain investigated (muscle recruitment changes triggered by previous episodes), an exploratory *post-hoc* subgroup analysis was conducted considering whether participants received training or not before the assessment. As reported in Fig 3, between-group differences were not identified in the subgroup of studies providing training before the assessment. On the other hand, when participants did not receive pre-assessment training or feedback, participants with rLBP showed a reduced thickness change of the TrA during a voluntary contraction.

#### Muscle timing

Evidence on muscle timing was obtained from four studies (*n* = 69) with a methodological quality ranging between poor and fair [13, 37, 51, 65]. Overall, very low quality of evidence (Table 4) supports delayed activity of TrA, OI and MF during postural and functional tasks in people with rLBP [13, 37, 51, 65]. Evidence from two studies on timing alterations of other trunk muscles was contrasting and characterised by very low quality evidence [51, 65].

All studies assessed the onset of axial muscle activity during limb movements while standing or during walking (SMDs are reported in S3 Table). The limited number of studies and the clinical heterogeneity across them did not allow the results to be synthesised quantitatively.

#### Spine kinematics

Seven studies (*n* = 136), with a methodological quality ranging between poor and fair, investigated kinematics of the spine in people with rLBP [34, 43, 47, 50, 62, 64, 66]. Spine kinematics was not investigated in people with rNP. Very low quality evidence (Table 4) supports reduced range of motion (ROM) of the thoracic and lumbar spine in people with rLBP during movements involving large excursion. Given the limitations of the evidence, meaningful conclusions could not be drawn for other kinematic features.

Decreased ROM of the thoracic and lumbar spine was reported in three studies; two including a full-range assessment (in standing and sitting) [47, 62] and one during functional tasks [43]. Other studies investigating small movements of the spine did not identify between-group differences [34, 50, 64, 66]. One study analysed the speed of spine motions during functional tasks (reaching) and reported slower trunk movements in people with rLBP [43]. Clinical and methodological heterogeneity across studies prevented a meta-analysis from being performed.

#### Proprioception

Very low quality evidence from one study (Table 4) supports impaired proprioception in people with rNP (*n* = 30) [43], whereas no differences were reported between control and rLBP people in two studies (*n* = 111) [61, 62]. Elsig et al. [33] using the cervicocephalic relocation test (in rotation) found greater repositioning error in people with rNP (SMD, 0.58; 95% CI: 0.20, 0.96). People with rLBP were assessed by Phillips [61, 62] through trunk flexion/extension movements in sitting and standing revealing no between-group differences.

#### Muscle properties

Despite findings of fatty infiltration and metabolic changes of the back extensors, only two studies investigated muscle properties in people with rLBP [45, 46]. Therefore, meaningful conclusions on muscle properties in people with rLBP could not be drawn. No studies investigated the properties of cervical muscles in people with rNP.

In one study, MF, erector spinae and psoas major were analysed with MRI and greater fatty infiltration was reported in people with rLBP [45]. However, when different lumbar levels were considered, results were conflicting. No differences were found regarding the cross-sectional area. The analysis of the transverse relaxation times under a resting condition (reflecting the molecular organisation of the tissue, and so the fibre metabolism) revealed lower values of the MF in people with rLBP (SMD, -2.08; 95% CI -4.09, -0.06) [46]. Opposite findings were reported after exercise (higher metabolic activity, SMD, 1.31; 95% CI 0.11, 2.51]). One study using ultrasound reported greater thickness of the TrA at rest in people with rLBP (SMD, 0.97; 95% CI 0.28, 1.66]) [58].

#### Neuromuscular performance

Six studies (*n* = 103) investigated trunk muscle strength and endurance during static and dynamic conditions [33, 39, 40, 46, 47, 55]. Methodological quality of individual studies ranged between poor and fair.

Overall, very low quality of evidence (Table 4) reported contrasting results on back muscle strength and endurance between people with and without rLBP. Evidence in people with rNP were too limited to draw meaningful conclusions.

Two studies assessed trunk flexor and extensor strength [40, 47]; no differences were observed between people with and without episodes of rLBP. The only exception was greater peak eccentric extension in healthy people compared to those with rLBP [47]. Back extensor endurance was assessed in three studies through the Sorensen test [40, 46, 55]; objective (time to task failure) and subjective (Borg scale) outcome measures were evaluated. A lower time to task failure in people with rLBP was reported in one study but not in another one [40, 55]. However, greater perceived effort was reported in the rLBP group [46]. One study investigating diaphragm strength and endurance found no force differences but greater fatigability between rLBP and healthy people [39]. Only one study assessed people with rNP and lower performance of the deep neck flexors was reported [33]. Strength and endurance of superficial neck muscles were not investigated.

## Discussion

This is the first rigorous systematic review to investigate neuromuscular adaptations specifically in people with recurrent spinal pain. When compared to a control group, very low quality of evidence supports greater co-contraction of abdominal and back superficial muscles, activity redistribution across lumbar extensor muscles, delayed onset of deep trunk muscles, and lumbar ROM reduction in people with rLBP. The paucity of evidence and inconsistency did not allow meaningful conclusions to be made for other outcome domains or for people with rNP.

Although the included studies investigated trunk muscle activity during different tasks, results supported three relevant findings in people with rLBP when compared to a control group. (i) Motor behaviour was characterised by a greater co-contraction of trunk muscles, with a bias toward preferentially recruiting superficial muscles; (ii) motor control changes involve a redistribution of activity within and between muscles; (iii) delayed activity of deep muscles (MF, TrA, and OI) during a postural task. In relation to changes in deeper muscle activity, deep multifidus (dMF) activity was reduced compared to the activity of the superficial fibres (sMF), with some authors arguing that the modulation of the latter is to compensate for the deficit of the former [56, 57]. Such mechanism could explain the inconsistency reported when MF is assessed with ultrasound or intramuscular EMG (iEMG), as iEMG allows to discriminate the behaviour of sMF and dMF while MF thickness change investigated with US could be biased by sMF [56, 57].

Different from the main findings, one study reported an overall reduction in the activity of trunk extensors [34]. Nevertheless, the task evaluated (trunk extension while standing) is challenging in people with a history of back pain and results should be interpreted with caution. In particular, a trunk extension is often a provocative movement for people with back pain [70], which might favour an inhibitory, rather than a co-contraction strategy. Another source of inconsistency across the literature was seen for the recruitment of TrA. However, it was possible to partially explore the heterogeneity via the *post-hoc* subgroup analysis. When participants were allowed training trials before testing, a learning effect could mask the actual impairment of TrA recruitment. Therefore, people with rLBP might show a reduced recruitment of the TrA, but this can be promptly addressed with a short period of training. Although previous works investigating people with cLBP supported this hypothesis showing that one session of motor control training was able to improve the recruitment of the TrA (increased thickness change between rest and drawing-in maneuver) [71, 72], the small number of included studies in this review and their poor quality did not allow us to draw a meaningful conclusion. High quality studies are warranted to investigate this hypothesis further. Regardless, evidence agrees that the timing of recruitment of deep trunk muscle is delayed in people with rLBP and this may therefore potentially play a role in the development of pain recurrence. Consistent onset delay was found in the TrA, OI and MF (short fibres) during limb movements [51, 57, 65]. Central nervous system adaptations (e.g. in motor planning), has been suggested as possible mechanism for these changes [73, 74]. Although participants were assessed when asymptomatic, previous painful episodes could have triggered motor strategies involving *en masse* recruitment possibly due to motor cortex reorganisation [75, 76]. For instance, motor region “smudging” was reported in people with a history of LBP and altered muscle recruitment during postural perturbations [74, 76, 77]. Specifically, the motor cortical areas of deep MF and longissimus erector spinae were overlapped in people with rLBP [76, 77]. Similar findings were reported also assessing neck muscles, but only in people with chronic NP [78].

Although affected by a very low quality of evidence, our findings are in accordance with those for people with chronic LBP [9, 16], and with contemporary theories of motor adaptation to pain [10, 14, 15]. Motor behaviour changes are heterogenous across individuals but appear to have the common goal of protection in the short term [10, 14, 15]. However, new strategies triggered by pain (e.g. redistributed activity, co-activation) can remain after symptom resolution and lead potentially to negative long-term consequences, such as sustained tissue loading, early fatigue, and poor inter-segmental motion [10, 14, 15]. In support of this, recent findings have shown that muscle activity changes triggered by experimental pain can last even after symptom resolution [79]. Longitudinal studies are needed to understand whether muscle activity changes are able to predict new painful episodes.

Regarding kinematics, very low quality of evidence supported a reduced ROM in people with rLBP. Although few studies were included, findings partially agree with kinematic changes reported in people with chronic LBP as synthesised by Laird et al [17]. Therefore, it seems that despite the absence of symptoms, kinematic changes also persist after an episode of LBP. Considering the gap in the literature, future studies are needed to assess spine kinematics in people with rNP. The limited number of studies, as well as their heterogeneity in the investigation of different muscles and spinal regions, did not allow us to draw conclusions on muscle properties and proprioception in people with recurrent spinal pain.

Although studies investigating neuromuscular performance in people with recurrent spinal pain were available, heterogeneity in their population, methodology, and results prevented meaningful conclusions. Overall, trunk muscle strength and endurance (when assessed objectively) did not differ between healthy and rLBP people, however the latter group reported greater perceived effort. This could be explained by psychological and/or biological factors. For example, self-efficacy and kinesiophobia were selected in a model to predict the time to task failure in people with rLBP [40]. On the other hand, D’Hooge et al. [46] hypothesised a change in the composition of back muscles resulting in a higher proportion of glycolytic fibres which would make muscles less efficient to sustain prolonged contractions [46]. However, results from studies investigating muscle fibre type proportions in people with chronic LBP are contrasting [80]. Despite the paucity and the very low quality of evidence, some axial muscles with a relevant role in spinal control showed poor performance in people with rNP and rLBP. For example, poor performance on the craniocervical flexion test (CCFT) was found in rNP people, showing similar values to a chronic NP population [11]. As it has been hypothesised in the lumbar spine, impaired activity of deep axial muscles could affect motor control and make people with rNP more prone to develop new painful episodes. A similar functional implication could be assigned to the endurance deficit of the diaphragm in people with rLBP [39]. In fact, this structure has a fundamental role to ensure spinal stability and regulate the intra-abdominal pressure during an effort [39]. Future research should focus on these aspects and evaluate impairments from a functional perspective. Moreover, the assessment of psychological features should be integrated to identify relevant associations.

### Strengths and limitations

This systematic review utilised a rigorous methodology, following a predefined and published protocol [20], as well as methodological checklists and GRADE approach to rate the overall quality of evidence of each outcome domain. Screening, quality assessment, and data extraction were conducted independently by two reviewers. The inclusion of different outcome domains ensured an extensive assessment of the literature investigating neuromuscular changes in people with recurrent spinal pain.

The use of subgroup analyses allowed us to explore the limitation arising from an inconsistent use of terminology across the literature and its associated risk to exclude relevant studies. However, from a methodological perspective, it was not possible to include studies in this review that mixed people with recurrent and chronic pain or studies considering a history of just one pain episode as a recurrent pattern. Eligibility criteria of future studies should adopt standardised definitions to allow comparison and generalisation of findings.

Other limitations of this review are the low quality evidence (both at a study and outcome level), as well as the small number and sample size of included studies. The clinical heterogeneity in people with spinal pain and the use of self-reported diagnosis for the recruitment of participants, negatively affected the quality of evidence. Therefore, findings from this review should be considered with caution, and integrated with those obtained from longitudinal studies.

Moreover, most of the included studies evaluated neuromuscular features in young adults, precluding findings to be generalised to middle-aged and older adult populations. Thus, studies investigating neuromuscular control in people with spinal pain from different age groups are required.

Finally, meaningful conclusions in people with recurrent neck pain were extremely limited because no evidence was available investigating muscle activity, spine kinematics, and muscle properties in this population.

### Clinical implications

The current findings reveal relevant clinical considerations. Most notably, the recovery from spinal pain symptoms does not directly correspond to the recovery of neuromuscular function and, in accordance with current theories on movement and pain [6–8], persistent neuromuscular adaptations could potentially impact on spinal pain recurrence. Nevertheless, a better understanding of the mechanisms underlying these neuromuscular adaptations and robust evidence for the relevance of these features for the development of future episodes of pain is needed. Therefore, future research including longitudinal designs are warranted to identify relevant predictors and unravel causal relationships.

## Conclusion

This review found very low level evidence supporting the existence of motor behaviour changes during a period of remission in people with rLBP. Motor strategies involving co-contraction, muscle activity redistribution, and delayed recruitment of deep axial muscles have been identified. There is evidence of limited ROM in the sagittal plane in people with rLBP. Investigation of other outcome domains concerning the neuromuscular system have received little attention, and there was very limited research on neuromuscular adaptations in people with rNP.

## Supporting information

S1 Table PRISMA checklist. (DOCX)

S2 Table Characteristics of excluded studies with reasons. (DOCX)

S3 Table Main findings. (DOCX)

S1 File Search strategy used in MEDLINE (OVID interface). (DOCX)

S2 File Contacted authors and studies. (DOCX)

S3 File Collated records. (DOCX)
