# Supplementary material for: Are neuromuscular adaptations present in people with recurrent spinal pain during a period of remission? a systematic review
Source: PLoS One. 2021 Apr 1;16(4):e0249220. doi: 10.1371/journal.pone.0249220 (PMC8016280; doi:10.1371/journal.pone.0249220)
Supplement: S2 Table — (DOCX) [file pone.0249220.s002.docx]

**S2 Table. Characteristics of excluded studies with reasons**

| **S2A Table. REASON 1.** Not recurrent spinal pain pattern (e.g. single episode of spinal pain, history of spinal pain) | |
| --- | --- |
| 1 | Akasaka K, Tamura A, Otsudo T, et al. Asymmetric trunk range of motion in college female students with low back pain. Man Ther. 2016;25:e158-e159 |
| 2 | Amabile AH, Bolte JH, Richter SD. Atrophy of gluteus maximus among women with a history of chronic low back pain. *PLoS One*. 2017;12(7):e0177008. Published 2017 Jul 17. doi:10.1371/journal.pone.0177008 |
| 3 | Artz, N.J., Phillips, D.R, Davey, C.A. and Hurley, M.V. (2003) Proprioception of the cervical spine in subjects with and without a history of neck pain. [in special issue: British Society for Rheumatology XX Annual General Meeting. A joint meeting with the Société Française de Rhumatologie, 1-4 April 2003, Manchester, UK] Rheumatology, 42, supplement 1, 146. |
| 4 | Babiolakis CS, Kuk JL, Drake JD. Differences in lumbopelvic control and occupational behaviours in female nurses with and without a recent history of low back pain due to back injury. *Ergonomics*. 2015;58(2):235‐245. doi:10.1080/00140139.2014.968635 |
| 5 | Burdorf A, van Riel M, van Wingerden JP, van Wingerden S, Snijders C. Isodynamic evaluation of trunk muscles and low-back pain among workers in a steel factory. *Ergonomics*. 1995;38(10):2107‐2117. doi:10.1080/00140139508925254 |
| 6 | Burke S, Lynch K, Moghul Z, Young C, Saviola K, Schenk R. The reliability of the cervical relocation test on people with and without a history of neck pain. *J Man Manip Ther*. 2016;24(4):210‐214. doi:10.1179/2042618615Y.0000000016 |
| 7 | Cherniack M, Dillon C, Erdil M, et al. Clinical and psychological correlates of lumbar motion abnormalities in low back disorders. Spine J. 2001;1(4):290‐298. doi:10.1016/s1529-9430(01)00104-8 |
| 8 | Chiou SY, Jeevathol A, Odedra A, Strutton PH. Voluntary activation of trunk extensors appears normal in young adults who have recovered from low back pain. Eur J Pain. 2015;19(10):1506‐1515. doi:10.1002/ejp.684 |
| 9 | Dubois JD, Cantin V, Piché M, Descarreaux M. Physiological and Psychological Predictors of Short-Term Disability in Workers with a History of Low Back Pain: A Longitudinal Study. PLoS One. 2016;11(10):e0165478. Published 2016 Oct 26. doi:10.1371/journal.pone.0165478 |
| 10 | Esola MA, McClure PW, Fitzgerald GK, Siegler S. Analysis of lumbar spine and hip motion during forward bending in subjects with and without a history of low back pain. *Spine (Phila Pa 1976)*. 1996;21(1):71‐78. doi:10.1097/00007632-199601010-00017 |
| 11 | Caroline Evans & William Oldreive (2000) A Study to Investigate Whether  Golfers with a History of Low Back Pain Show a Reduced Endurance of Transversus Abdominis,Journal of Manual & Manipulative Therapy, 8:4, 162-174, DOI: 10.1179/jmt.2000.8.4.162 |
| 12 | Fall MP. Electromyographic analysis of the lumbar erector spinae muscles: Influence of position, a history of low back pain, gender and muscle location on fatigue and recovery. PhD Thesis. 2001; University of Connecticut |
| 13 | Grabiner MD, Jeziorowski JJ. Isokinetic trunk extension discriminates uninjured subjects from subjects with previous low back pain. Clin Biomech (Bristol, Avon). 1992;7(4):195‐200. doi:10.1016/S0268-0033(92)90001-K |
| 14 | Grenier SG, McGill SM. When exposed to challenged ventilation, those with a history of LBP increase spine stability relatively more than healthy individuals. Clin Biomech (Bristol, Avon). 2008;23(9):1105‐1111. doi:10.1016/j.clinbiomech.2008.06.010 |
| 15 | Haddas R, Yang J, Lieberman I. Effects of volitional spine stabilization on lifting task in recurrent low back pain population. *Eur Spine J*. 2016;25(9):2833‐2841. doi:10.1007/s00586-016-4586-1 |
| 16 | Haddas R, Sawyer SF, Sizer PS Jr, Brooks T, Chyu MC, James CR. Effects of Volitional Spine Stabilization and Lower Extremity Fatigue on Trunk Control During Landing in Individuals With Recurrent Low Back Pain. *J Orthop Sports Phys Ther*. 2016;46(2):71‐78. doi:10.2519/jospt.2016.6048 |
| 17 | Ham YW, Kim DM, Baek JY, Lee DC, Sung PS. Kinematic analyses of trunk stability in one leg standing for individuals with recurrent low back pain. *J Electromyogr Kinesiol*. 2010;20(6):1134‐1140. doi:10.1016/j.jelekin.2010.05.011 |
| 18 | Hernandez A, Gross K, Gombatto S. Differences in lumbar spine and lower extremity kinematics during a step down functional task in people with and people without low back pain. *Clin Biomech (Bristol, Avon)*. 2017;47:46‐52. doi:10.1016/j.clinbiomech.2017.05.012 |
| 19 | Hodges P, van den Hoorn W, Dawson A, Cholewicki J. Changes in the mechanical properties of the trunk in low back pain may be associated with recurrence. *J Biomech*. 2009;42(1):61‐66. doi:10.1016/j.jbiomech.2008.10.001 |
| 20 | Hultman G, Nordin M, Saraste H, Ohlsèn H. Body composition, endurance, strength, cross-sectional area, and density of MM erector spinae in men with and without low back pain. *J Spinal Disord*. 1993;6(2):114‐123. |
| 21 | Johnson EN, Thomas JS. Effect of hamstring flexibility on hip and lumbar spine joint excursions during forward-reaching tasks in participants with and without low back pain. *Arch Phys Med Rehabil*. 2010;91(7):1140‐1142. doi:10.1016/j.apmr.2010.04.003 |
| 22 | Jones SL. Postural control impairment characteristics of chronic, recurrent low back pain: an investigation of automatic postural responses and sit-to-stand movements. PhD Thesis. 2010; McGill University |
| 23 | Kauther MD, Piotrowski M, Hussmann B, Lendemans S, Wedemeyer C. Cervical range of motion and strength in 4,293 young male adults with chronic neck pain. *Eur Spine J*. 2012;21(8):1522‐1527. doi:10.1007/s00586-012-2369-x |
| 24 | Larmer A, Sheeran L, Sparkes V. Lumbar spine angles in subjects with a history of flexion-related back pain when performing a typing task. Spine Journal. 2016;1:S76-S77 |
| 25 | Larsen LH, Brogner HM, Oestergaard GB. Disturbed muscular adaptation and changed motor strategies in recurrent low back pain patients during stair walking after experimental back-muscle fatigue. Physiotherapy. 2015;1:eS834 |
| 26 | Latimer J, Maher CG, Refshauge K, Colaco I. The reliability and validity of the Biering-Sorensen test in asymptomatic subjects and subjects reporting current or previous nonspecific low back pain. *Spine (Phila Pa 1976)*. 1999;24(20):2085‐2090. doi:10.1097/00007632-199910150-00004 |
| 27 | Lee H, Nicholoson LL, Adams RD, Bae SS. Body chart pain location and side-specific physical impairment in subclinical neck pain. *J Manipulative Physiol Ther*. 2005;28(7):479‐486. doi:10.1016/j.jmpt.2005.07.004 |
| 28 | Lee H, Nicholson LL, Adams RD. Cervical range of motion associations with subclinical neck pain. Spine (Phila Pa 1976). 2004;29(1):33‐40. doi:10.1097/01.BRS.0000103944.10408.BA |
| 29 | Lee H, Nicholson LL, Adams RD, Bae SS. Proprioception and rotation range sensitization associated with subclinical neck pain. *Spine (Phila Pa 1976)*. 2005;30(3):E60‐E67. doi:10.1097/01.brs.0000152160.28052.a2 |
| 30 | Lee JH, Ooi Y, Nakamura K. Measurement of muscle strength of the trunk and the lower extremities in subjects with history of low back pain. *Spine (Phila Pa 1976)*. 1995;20(18):1994‐1996. doi:10.1097/00007632-199509150-00006 |
| 31 | Tsai YS, Sell TC, Smoliga JM, Myers JB, Learman KE, Lephart SM. A comparison of physical characteristics and swing mechanics between golfers with and without a history of low back pain. *J Orthop Sports Phys Ther*. 2010;40(7):430‐438. doi:10.2519/jospt.2010.3152 |
| 32 | Liew X, Allison G, Gibson W. Influence of attentional focus on spinal reposition performance in people with and without a history of low back pain. Journal of Science and Medicine in Sport. 2011;1:e97-e98 |
| 33 | Lourenço AS, Lameiras C, Silva AG. Neck Flexor and Extensor Muscle Endurance in Subclinical Neck Pain: Intrarater Reliability, Standard Error of Measurement, Minimal Detectable Change, and Comparison With Asymptomatic Participants in a University Student Population. *J Manipulative Physiol Ther*. 2016;39(6):427‐433. doi:10.1016/j.jmpt.2016.05.005 |
| 34 | Masaki M, Tateuchi H, Koyama Y, Sakuma K, Otsuka N, Ichihashi N. Back muscle activity and sagittal spinal alignment during quadruped upper and lower extremity lift in young men with low back pain history. *Gait Posture*. 2018;66:221‐227. doi:10.1016/j.gaitpost.2018.09.002 |
| 35 | Mattison MC, Ross NA. Comparing fatigue responses between healthy individuals and asymptomatic low back pain sufferers - implications for return-to-work: a pilot study. Ergonomics SA. 2016;28(2):2-18 |
| 36 | Mazis N. Does a history of non specific low back pain influence electromyographic activity of the erector spinae muscle group during functional movements? J Nov Physiother. 2014;4(4):1-11 |
| 37 | McClure PW, Esola M, Schreier R, Siegler S. Kinematic analysis of lumbar and hip motion while rising from a forward, flexed position in patients with and without a history of low back pain. *Spine (Phila Pa 1976)*. 1997;22(5):552‐558. doi:10.1097/00007632-199703010-00019 |
| 38 | McGill S, Grenier S, Bluhm M, Preuss R, Brown S, Russell C. Previous history of LBP with work loss is related to lingering deficits in biomechanical, physiological, personal, psychosocial and motor control characteristics. *Ergonomics*. 2003;46(7):731‐746. doi:10.1080/0014013031000090134 |
| 39 | Mellin G. Decreased joint and spinal mobility associated with low back pain in young adults. *J Spinal Disord*. 1990;3(3):238‐243. |
| 40 | Murakami T, Sakuraba K, Nagai K. Comparison of changes in the transversus abdominis and neighboring fascia in subjects with and without a history of low back pain using ultrasound imaging. J Phys Ther Sci, 2011, 23: 317–322. |
| 41 | Nagai T, Abt JP, Sell TC, et al. Lumbar spine and hip flexibility and trunk strength in helicopter pilots with and without low back pain history. *Work*. 2015;52(3):715‐722. doi:10.3233/WOR-152192 |
| 42 | Noormohammadpour P, Mirzaei S, Moghadam N, Mansournia MA, Kordi R. Comparison of lateral abdominal muscle thickness in young male soccer players with and without low back pain. *Int J Sports Phys Ther*. 2019;14(2):273‐281. |
| 43 | Pacheco J, Raimundo J, Santos F, et al. Forward head posture is associated with pressure pain threshold and neck pain duration in university students with subclinical neck pain. *Somatosens Mot Res*. 2018;35(2):103‐108. doi:10.1080/08990220.2018.1475352 |
| 44 | Paulus I, Brumagne S. Altered interpretation of neck proprioceptive signals in persons with subclinical recurrent neck pain. *J Rehabil Med*. 2008;40(6):426‐432. doi:10.2340/16501977-0189 |
| 45 | Quan G, McCreesh K. A study to determine whether rowers with a history of low back pain shows a reduced endurance time of transversus abdominus compared to rowers without such a history. Physiotherapy Ireland. 2007;28(1):48 |
| 46 | Ratzon NZ, Jarus T, Catz A. The relationship between work function and low back pain history in occupationally active individuals. *Disabil Rehabil*. 2007;29(10):791‐796. doi:10.1080/09638280600919681 |
| 47 | Reis F, Macedo AR, Jatoba DR, Lota FP, Prazeres G, Souza MRDS. Cervical joint position sense: A comparison between neck pain and asymptomatic student volunteers. 2011. Physiotherapy (United Kingdom);1:eS1042 |
| 48 | Roussel N, De Kooning M, Schutt A, et al. Motor control and low back pain in dancers. *Int J Sports Med*. 2013;34(2):138‐143. doi:10.1055/s-0032-1321722 |
| 49 | Seay JF, Sauer SG, Frykman PN, Roy TC. A history of low back pain affects pelvis and trunk mechanics during a sustained lift/lower task. *Ergonomics*. 2013;56(6):944‐953. doi:10.1080/00140139.2013.781234 |
| 50 | Seay JF, Sauer SG, Patel T, Roy TC. A history of low back pain affects pelvis and trunk coordination during a sustained manual materials handling task. *J Sport Health Sci*. 2016;5(1):52‐60. doi:10.1016/j.jshs.2016.01.011 |
| 51 | Sihvonen T, Lindgren KA, Airaksinen O, Manninen H. Movement disturbances of the lumbar spine and abnormal back muscle electromyographic findings in recurrent low back pain. *Spine (Phila Pa 1976)*. 1997;22(3):289‐295. doi:10.1097/00007632-199702010-00012 |
| 52 | Smith K, Hall T, Robinson K. The influence of age, gender, lifestyle factors and sub-clinical neck pain on the cervical flexion-rotation test and cervical range of motion. *Man Ther*. 2008;13(6):552‐559. doi:10.1016/j.math.2007.07.005 |
| 53 | Sperry MM, Phillips ATM, McGregor AH. Lower back pain and healthy subjects exhibit distinct lower limb perturbation response strategies: A preliminary study. *J Back Musculoskelet Rehabil*. 2019;32(1):27‐35. doi:10.3233/BMR-170808 |
| 54 | Sung P, Jo H, Song A. Kinematic analyses of trunk stability in individuals with recurrent low back pain. 2012. Pain Practice;2012(1):196 |
| 55 | Sung PS, Danial P, Lee DC. Comparison of the different kinematic patterns during lateral bending between subjects with and without recurrent low back pain. *Clin Biomech (Bristol, Avon)*. 2016;38:50‐55. doi:10.1016/j.clinbiomech.2016.08.006 |
| 56 | Sung PS, Leininger PM. A kinematic and kinetic analysis of spinal region in subjects with and without recurrent low back pain during one leg standing. *Clin Biomech (Bristol, Avon)*. 2015;30(7):696‐702. doi:10.1016/j.clinbiomech.2015.05.003 |
| 57 | Takahashi I, Kikuchi S, Sato K, Iwabuchi M. Effects of the mechanical load on forward bending motion of the trunk: comparison between patients with motion-induced intermittent low back pain and healthy subjects. *Spine (Phila Pa 1976)*. 2007;32(2):E73‐E78. doi:10.1097/01.brs.0000252203.16357.9a |
| 58 | Teng CC, Chai H, Lai DM, Wang SF. Cervicocephalic kinesthetic sensibility in young and middle-aged adults with or without a history of mild neck pain. *Man Ther*. 2007;12(1):22‐28. doi:10.1016/j.math.2006.02.003 |
| 59 | Tsai Y. Biomechanical and physical characteristics of trunk and hip in golfers with and without low back pain. PhD Thesis. 2005;University of Pittsburgh |
| 60 | Tsao H, Galea MP, Hodges PW. Reorganization of the motor cortex is associated with postural control deficits in recurrent low back pain. *Brain*. 2008;131(Pt 8):2161‐2171. doi:10.1093/brain/awn154 |
| 61 | Vad VB, Bhat AL, Basrai D, Gebeh A, Aspergren DD, Andrews JR. Low back pain in professional golfers: the role of associated hip and low back range-of-motion deficits. *Am J Sports Med*. 2004;32(2):494‐497. doi:10.1177/0363546503261729 |
| 62 | Wattananon P, Intawachirarat N, Cannella M, Sung W, Silfies SP. Reduced instantaneous center of rotation movement in patients with low back pain. *Eur Spine J*. 2018;27(1):154‐162. doi:10.1007/s00586-017-5054-2 |
| 63 | Zabihhosseinian M, Holmes MW, Howarth S, Ferguson B, Murphy B. Neck muscle fatigue differentially alters scapular and humeral kinematics during humeral elevation in subclinical neck pain participants versus healthy controls. J Electromyogr Kinesiol. 2017;33:73‐82. doi:10.1016/j.jelekin.2017.02.002 |
| 64 | Zabihhosseinian M, Holmes MW, Ferguson B, Murphy B. Neck muscle fatigue alters the cervical flexion relaxation ratio in sub-clinical neck pain patients. *Clin Biomech (Bristol, Avon)*. 2015;30(5):397‐404. doi:10.1016/j.clinbiomech.2015.03.020 |
| 65 | Sung PS. A kinematic analysis for shoulder and pelvis coordination during axial trunk rotation in subjects with and without recurrent low back pain. Gait Posture. 2014;40(4):493‐498. doi:10.1016/j.gaitpost.2014.06.001 |
| 66 | Helewa A, Goldsmith CH, Smythe HA. Measuring abdominal muscle weakness in patients with low back pain and matched controls: a comparison of 3 devices. *J Rheumatol*. 1993;20(9):1539‐1543. |
| 67 | Masaki M, Ikezoe T, Yanase K, et al. Association of Pain History and Current Pain With Sagittal Spinal Alignment and Muscle Stiffness and Muscle Mass of the Back Muscles in Middle-aged and Elderly Women. *Clin Spine Surg*. 2019;32(7):E346‐E352. doi:10.1097/BSD.0000000000000793 |
| 68 | Farid B. Multisensory Integration in Subclinical Neck Pain. Master’s Thesis. 2016; University of Ontario Institute of Technology |
| 69 | Haddas R, Samocha Y, Yang J. Effects of Volitional Spine Stabilization on Trunk Control During Asymmetric Lifting Task in Patients With Recurrent Low Back Pain. Global Spine J. 2019 Oct 28. doi: 10.1177/2192568219885898. |
| 70 | Martinez-Valdes E, Wilson F, Fleming N, McDonnell SJ, Horgan A, Falla D. Rowers with a recent history of low back pain engage different regions of the lumbar erector spinae during rowing. J Sci Med Sport. 2019 Nov;22(11):1206-1212. doi: 10.1016/j.jsams.2019.07.007. |
| 71 | Moreno-Pérez V, López-Valenciano A, Ayala F, Fernandez-Fernandez J, Vera-Garcia FJ. Comparison of hip extension and rotation ranges of motion in young elite tennis players with and without history of low back pain. J Back Musculoskelet Rehabil. 2019;32(4):629-638. doi: 10.3233/BMR-181296. PMID: 30614792. |
| 72 | Nandlall N, Rivaz H, Rizk A, Frenette S, Boily M, Fortin M. The effect of low back pain and lower limb injury on lumbar multifidus muscle morphology and function in university soccer players. BMC Musculoskelet Disord. 2020 Feb 12;21(1):96. doi: 10.1186/s12891-020-3119-6. |
| 73 | Oshikawa T, Kaneoka K, Morimoto Y, Akuzawa H. Comparison of Lumbar Kinematics with a History of Low Back Pain During Baseball Hitting. Int J Sports Med. 2020 Feb;41(2):119-127. doi: 10.1055/a-1014-3041. Epub 2020 Jan 7. |
| 74 | Shojaei I, Salt EG, Bazrgari B. A prospective study of lumbo-pelvic coordination in patients with non-chronic low back pain. J Biomech. 2020 Mar 26;102:109306. doi: 10.1016/j.jbiomech.2019.07.050. |

| **S2B Table. REASON 2.** Limited period of “no spinal pain” in the control group (e.g. “no history of back pain in the previous 6 months”) | |
| --- | --- |
| 1 | Ang B, Linder J, Harms-Ringdahl K. Neck strength and myoelectric fatigue in fighter and helicopter pilots with a history of neck pain. *Aviat Space Environ Med*. 2005;76(4):375‐380. (6 months) |
| 2 | Crosbie J, de Faria Negrão Filho R, Nascimento DP, Ferreira P. Coordination of spinal motion in the transverse and frontal planes during walking in people with and without recurrent low back pain. Spine (Phila Pa 1976). 2013;38(5):E286‐E292. doi:10.1097/BRS.0b013e318281de28 (6 months) |
| 3 | Hedayati R, Kahrizi S, Parnianpour M, Bahrami F, Kazemnejad A, Mobini B. The study of the variability of anticipatory postural adjustments in patients with recurrent non-specific low back pain. *J Back Musculoskelet Rehabil*. 2014;27(1):33‐40. doi:10.3233/BMR-130416 (1 year) |
| 4 | Lecompte J, Maisetti O, Guillaume A, Skalli W, Portero P. Neck strength and EMG activity in fighter pilots with episodic neck pain. *Aviat Space Environ Med*. 2008;79(10):947‐952. doi:10.3357/asem.2167.2008 |
| 5 | Leinonen V, Kankaanpää M, Vanharanta H, Airaksinen O, Hänninen O. Back and neck extensor loading and back pain provocation in urban bus drivers with and without low back pain. *Pathophysiology*. 2005;12(4):249‐255. doi:10.1016/j.pathophys.2005.09.004 |
| 6 | Nagai T, Abt JP, Sell TC, et al. Neck proprioception, strength, flexibility, and posture in pilots with and without neck pain history. *Aviat Space Environ Med*. 2014;85(5):529‐535. doi:10.3357/asem.3874.2014 (1 year) |
| 7 | Rowley KM, Smith JA, Kulig K. Reduced Trunk Coupling in Persons With Recurrent Low Back Pain Is Associated With Greater Deep-to-Superficial Trunk Muscle Activation Ratios During the Balance-Dexterity Task. *J Orthop Sports Phys Ther*. 2019;49(12):887‐898. doi:10.2519/jospt.2019.8756 |
| 8 | Lee SP, Dinglasan V, Duong A, Totten R, Smith JA. Individuals With Recurrent Low Back Pain Exhibit Significant Changes in Paraspinal Muscle Strength After Intramuscular Fine Wire Electrode Insertion. PM R. 2020 Aug;12(8):775-782. doi: 10.1002/pmrj.12284. Epub 2019 Dec 28. PMID: 31707770. |
| 9 | Rowley KM, Engel T, Kulig K. Trunk and hip muscle activity during the Balance-Dexterity task in persons with and without recurrent low back pain. J Electromyogr Kinesiol. 2020 Feb;50:102378. doi: 10.1016/j.jelekin.2019.102378. Epub 2019 Nov 20. |
| 10 | Rowley KM, Winstein CJ, Kulig K. Persons in remission from recurrent low back pain alter trunk coupling under dual-task interference during a dynamic balance task. Exp Brain Res. 2020 Apr;238(4):957-968. doi: 10.1007/s00221-020-05772-4. |
| 11 | Schouppe S, Clauwaert A, Van Oosterwijck J, Van Damme S, Palmans T, Wiersema JR, Sanchis-Sanchéz E, Danneels L. Does experimentally induced pain-related fear influence central and peripheral movement preparation in healthy people and patients with low back pain? Pain. 2020 Jun;161(6):1212-1226. doi: 10.1097/j.pain.0000000000001813. |

| **S2C Table. REASON 3.** People with recurrent or chronic spinal pain were included in the same group. | |
| --- | --- |
| 1 | Arab AM, Ghamkhar L, Emami, M. Altered pattern of the lumbo-pelvic muscles activity during prone hip extension in women with low back pain. Physiotherpay. 2011;1:eS79-eS80 |
| 2 | Barker I. Alterations in neck muscle performance and proprioception with fatique, altered posture and recurrent neck pain. Master Thesis. 2011; University of Ontario Institute of Technology |
| 3 | Chimenti RL, Scholtes SA, Van Dillen LR. Activity characteristics and movement patterns in people with and people without low back pain who participate in rotation-related sports. J Sport Rehabil. 2013;22(3):161‐169. doi:10.1123/jsr.22.3.161 |
| 4 | Jacobs JV, Henry SM, Jones SL, Hitt JR, Bunn JY. A history of low back pain associates with altered electromyographic activation patterns in response to perturbations of standing balance. J Neurophysiol. 2011;106(5):2506‐2514. doi:10.1152/jn.00296.2011 |
| 5 | Lee HY, Wang JD, Yao G, Wang SF. Association between cervicocephalic kinesthetic sensibility and frequency of subclinical neck pain. *Man Ther*. 2008;13(5):419‐425. doi:10.1016/j.math.2007.04.001 |
| 6 | Mok NW, Brauer SG, Hodges PW. Failure to use movement in postural strategies leads to increased spinal displacement in low back pain. *Spine (Phila Pa 1976)*. 2007;32(19):E537‐E543. doi:10.1097/BRS.0b013e31814541a2 |
| 7 | Scholtes SA. The effect of limb movement on the lumbopelvic region in people with low back pain. PhD Thesis. 2009. Washington University in St. Louis |

| **S2D Table. REASON 4.** No outcome of interest | |
| --- | --- |
| 1 | Hart JM, Beazell J, Ingersoll CD, Weltman A, Hertel J. Exercise-Related Postural Control Deficits in Individuals with Recurrent Low Back Pain. Athletic Training and Sports Health Care. 2011;3(3):118-124 |
| 2 | Park WH, Kim YH, Lee TR, Sung PS. Factors affecting shoulder-pelvic integration during axial trunk rotation in subjects with recurrent low back pain. *Eur Spine J*. 2012;21(7):1316‐1323. doi:10.1007/s00586-012-2280-5 |

| **S2E Table. REASON 5.** Not English language | |
| --- | --- |
| 1 | Morita AK, Marques NR, Navega MT. Bilateral analysis of the anticipatory trunk muscle response in recurrent low back pain. ConScientiae Saude. 2018;17(2):127-134 |

| **S2F Table. REASON 6.** Radiating pain | |
| --- | --- |
| 1 | Sánchez-Zuriaga D, López-Pascual J, Garrido-Jaén D, García-Mas MA. A comparison of lumbopelvic motion patterns and erector spinae behavior between asymptomatic subjects and patients with recurrent low back pain during pain-free periods. *J Manipulative Physiol Ther*. 2015;38(2):130‐137. doi:10.1016/j.jmpt.2014.11.002 |

| **S2G Table. REASON 7.** Longitudinal design | |
| --- | --- |
| 1 | Silfies SP, Cholewicki J, Reeves NP, Greene HS. Lumbar position sense and the risk of low back injuries in college athletes: a prospective cohort study. *BMC Musculoskelet Disord*. 2007;8:129. Published 2007 Dec 31. doi:10.1186/1471-2474-8-129 |

| **S2H Table. REASON 8.** Eligibility criteria not clear and no reply from authors | |
| --- | --- |
| 1 | He J, Nakajima T, Espinoza Orias AA, An HS, Inoue N. Characterizing lumbar multifidus fatty infiltration with MRI: Is there a correct region of interest? Journal of Orthopaedic Research. 2016;34(1) |
| 2 | Hides JA, Boughen CL, Stanton WR, Strudwick MW, Wilson SJ. A magnetic resonance imaging investigation of the transversus abdominis muscle during drawing-in of the abdominal wall in elite Australian Football League players with and without low back pain. *J Orthop Sports Phys Ther*. 2010;40(1):4‐10. doi:10.2519/jospt.2010.3177 |
| 3 | Sung PS, Danial P. Analysis of relative kinematic index with normalized standing time between subjects with and without recurrent low back pain. *Eur Spine J*. 2017;26(2):518‐527. doi:10.1007/s00586-016-4727-6 |
| 4 | Sung PS, Danial P. Trunk Reaction Time and Kinematic Changes Following Slip Perturbations in Subjects with Recurrent Low Back Pain [published correction appears in Ann Biomed Eng. 2019 Jan;47(1):333]. *Ann Biomed Eng*. 2018;46(3):488‐497. doi:10.1007/s10439-017-1972-8 |
| 5 | Sung PS, Danial P. Trunk sway response to consecutive slip perturbations between subjects with and without recurrent low back pain [published correction appears in Musculoskelet Sci Pract. 2018 Dec;38:155]. *Musculoskelet Sci Pract*. 2018;33:84‐89. doi:10.1016/j.msksp.2017.12.005 |
| 6 | Sung PS, Lammers AR, Danial P. Different parts of erector spinae muscle fatigability in subjects with and without low back pain. *Spine J*. 2009;9(2):115‐120. doi:10.1016/j.spinee.2007.11.011 |
| 7 | Sung PS, Maxwell MJ. Kinematic chain reactions on trunk and dynamic postural steadiness in subjects with recurrent low back pain. *J Biomech*. 2017;59:109‐115. doi:10.1016/j.jbiomech.2017.06.001 |

| **S2I Table. REASON 9.** No full-text and no reply from authors | |
| --- | --- |
| 1 | Lee JH, Ooi Y, Hoshino Y, Nakamura K. Measurement of muscle cross-sectional area of the trunk and the lower extremities in subjects with history of low back pain. JNMS. 1996;4(4):131-136 |

| **S2J Table. REASON 10.** Recurrent spinal pain group assessed while symptomatic | |
| --- | --- |
| 1 | Qu N, Graven-Nielsen T, Lindstrøm R, Blogg Andersen Dc V, Hirata RP. Recurrent neck pain patients exhibit altered joint motion pattern during cervical flexion and extension movements. Clin Biomech (Bristol, Avon). 2020 Jan;71:125-132. doi: 10.1016/j.clinbiomech.2019.10.026. |
