# Supplementary material for: Are neuromuscular adaptations present in people with recurrent spinal pain during a period of remission? a systematic review
Source: PLoS One. 2021 Apr 1;16(4):e0249220. doi: 10.1371/journal.pone.0249220 (PMC8016280; doi:10.1371/journal.pone.0249220)
Supplement: S3 Table — (DOCX) [file pone.0249220.s003.docx]

**S3 Table. Main findings**

| Study | Task | Outcome measure | Body region | Control  mean±SD (n) | Rec spinal pain  mean±SD (n) | SMD (±95% CI) | NOS  (0/9) |
| --- | --- | --- | --- | --- | --- | --- | --- |
| **Muscle activity -** *Amplitude/Recruitment* | | | | | | | |
| Claus et al.[42] | Sitting in different postures   - Short lordosis [SL] - Long lordosis [LL] - Flat [F] | Peak RMS (% of MVC) | Longissimus T11  Iliocostalis T11  Iliocostalis L2  Deep MF L4  Sup MF L4  TrA  OI/TrA  OE  RA | 12.5 ± 7.9 (14) [LL]  3.0 ± 2.3 [SL]  3.5 ± 1.99 [SL]  16.8 ± 6.8 [SL]  10.9 ± 4.5 [SL]  4.3 ± 2.86 [SL]  4.0 ± 1.65 [SL]  3.4 ± 1.91 [LL]  0.9 ± 0.75 [SL] | 41.2 ± 17.3 (10) [LL]  8.8 ± 4.83 [SL]  8.1 ± 4.83 [SL]  17.5 ± 9.8 [SL]  13.5 ± 8.47 [LL]  11.6 ± 7.77[LL]  1.4 ± 1.33 [F]  2.2 ± 1.75 [F]  2.3 ± 2.38 [SL] | **2.27 (1.24 to 3.31)^a^**  **1.63 (0.70 to 2.57) ^a^**  **1.16 (0.29 to 2.04) ^a^**  0.09 (-0.73 to 0.90)  0.40 (-0.42 to 1.22)  **1.34 (0.45 to 2.24) ^a^**  -1.70 (-2.65 to -0.76)  -0.65 (-1.48 to 0.18)  0.86 (0.01 to 1.71) | **2** |
| D’hooge et al.[44] | Rapid trunk flexion:   - NO load - Load - NO load - Load | Summed RMS-EMG  (MVIC normalised) | Abdominal muscles  Paraspinal muscles | - 1. ± 0.92 (14)   1.18 ± 0.92  2.32 ± 1.23  2.64 ± 1.18 | 0.78 ± 0.8 (11)  0.93 ± 0.8  2.61 ± 0.91  2.94 ± 0.92 | -0.26 (-1.06 to 0.53)  -0.29 (-1.08 to 0.51)  0.26 (-0.53 to 1.06)  0.28 (-0.51 to 1.07) | **2** |
| Ferreira et al.[48] | Knee flexion and extension in supine position (load=7.5% body weight) | Muscle activation ratio  % RMS increase (normalised to MVC) | TrA  OI  OE  TrA  OI  OE | 1.19 ± 0.18 (10)  1.08 ± 0.10  1.01 ± 0.06  13 ± 8  8 ± 6  5 ± 5 | 1.04 ± 0.07 (10)  1.03 ± 0.02  1.01 ± 0.03  5 ± 4  4 ± 2  10 ± 11 | **-1.05 (-2.0 to -0.10) ^a^**  -0.69 (-1.60 to 0.20)  0 (-0.88 to 0.88)  **-1.27(-2.23 to -0.30) ^a^**  -0.90 (-1.81 to 0.03)  0.59 (-0.31 to 1.48) | **2** |
| Gorbet et al.[49] | ADM (supine position)  Quadruped exercise | Muscle activation ratio | TrA | 1.52 ± 0.27 (30)  1.43 ± 0.26 | 1.60 ± 0.32 (30)  1.49 ± 0.38 | 0.27 (-0.24 to 0.78)  0.18 (-0.32 to 0.69) | **3** |
| Himes et al.[38] | Side-bridge level:   - 1 - 2 - 3 - 4 - 5 | Muscle activation ratio  (exercise/rest) | TrA | 1.57 ± 0.40 (24)  1.52 ± 0.63  1.43 ± 0.42  1.46 ± 0.49  1.24 ± 0.57 | 1.64 ± 0.52 (23)  1.68 ± 0.70  1.53 ± 0.55  1.51 ± 0.58  1.38 ± 0.68 | 0.15 (-0.42 to 0.72)  0.24 (-0.33 to 0.81)  0.20 (-0.37 to 0.78)  0.09 (-0.48 to 0.67)  0.22 (-0.35 to 0.80) | **3** |
| Larsen et al.[34] | Step up (Ascent)  Step down (Descent) | RMS-EMG (%MVC) | Iliocostalis  Longissimus  MF  RA  OE  OI  Glut medius  Glut max  Iliocostalis  Longissimus  MF  RA  OE  OI  Glut medius  Glut max | - (25)  -  -  -  -  -  -  -  - (25)  -  -  -  -  -  -  - | - (25)  -  -  -  -  -  -  -  - (25)  -  -  -  -  -  -  - | **rLBP > Control ^a^**  **rLBP > Control ^a^**  **rLBP > Control ^a^**  **rLBP > Control ^a^**  **rLBP > Control ^a^**  **rLBP > Control ^a^**  NS  NS  **rLBP > Control ^a^**  **rLBP > Control ^a^**  **rLBP > Control ^a^**  **rLBP > Control ^a^**  **rLBP > Control ^a^**  **rLBP > Control ^a^**  NS  NS | **3**  *(continued on next page)* |
| MacDonald et al.[56] | Trunk loading   - Predictable - Unpredictable | Amplitude | Deep MF  Superficial MF  Deep MF  Superficial MF | (n=14) | (n=13) | rLBP < Control  NS  rLBP < Control  rLBP < Control | **2** |
| MacDonald et al.[57] | ASLR  CLR  PSLR | % Muscle thickness change | Lumbar MF | 2.99 ± 8.4 (10)  2.47 ± 9.0  17.04 ± 22.8 | 4.55 ± 13.5 (8)  4.16 ± 14.4  31.62 ± 36.0 | 0.14 (-0.79 to 1.07)  0.15 (-0.79 to 1.08)  0.49 (-0.45 to 1.44) | **2** |
| Nagar et al.[58] | ADM during   - Quiet standing - Loaded reaching | Muscle activation ratio (exercise/rest) | TrA | 1.40 ± 0.30 (18)  1.50 ± 0.35 | 1.36 ± 0.30 (18)  1.42 ± 0.35 | -0.13 (-0.78 to 0.52)  -0.23 (-0.88 to 0.43) | **3** |
| Park et al.[59]      Park et al.[60] | Trunk loading, direction:   - Flexion - Extension - Lat flex right   Three sit postures   - Slump - Flat - Short lordotic | RMS, % MVC  RMS, % MVC | **PM-v**  QL-p  ES  OE  OI/TrA  PM-t  QL-p  ES  OE  OI/TrA  **PM-v**  **PM-t**  **QL-a**  ES  OE  OI/TrA  PM-v  QL-p  ES  OE  OI/TrA  **PM-v**  **QL-p**  ES  OE  OI/TrA  PM-v  **QL-p**  ES  OE  OI/TrA | **15.0 ± 5.6** (9)  4.2 ± 5.0  2.5 ± 5.6  9.2 ± 5.6  6.9 ± 5.6  19.5 ± 4.7  2.7 ± 4.9  9.6 ± 4.9  1.6 ± 4.9  4.1 ± 4.9  **35.3 ± 10.5**  **27.9 ± 10.5**  **13.52 ± 10.5**  7.17 ± 10.5  3.03 ± 10.2  9.38 ± 10.2  2.0 ± 1.2  0.6 ± 1.2  1.1 ± 1.2  1.3 ± 1.3  1.4 ± 1.1  **6.7 ± 2.7**  **0.9 ± 2.7**  1.3 ± 2.7  1.4 ± 2.8  2.2 ± 2.7  7.8 ± 2.8  **1.0 ± 2.7**  3.2 ± 2.8  1.9 ± 2.8  3.4 ± 2.7 | rLBP low \| rLBP high  n=10 | **1.53 (0.51 to 2.56) ^a^**  **1.51 (0.49 to 2.53) ^a^**  **-1.42 (-2.43 to -0.42)^a^**  **2.11 (0.99 to 3.23) ^a^**  *(continued on next page)*  **1.78 (0.71 to 2.84)^a^**  **2.02 (0.91 to 3.12)^a^**  **2.76 (1.50 to 4.02)^a^** | **2**  **2** |
|  |  |  |  |  | **25.8 ± 8.1** \| 5.0 ± 7.5  5.6 ± 8.9 \| 2.2 ± 7.5  2.2 ± 8.3 \| 4.2 ± 7.8  20.0 ± 7.8 \| 8.6 ± 8.1  6.4 ± 7.8 \| 7.5 ± 7.5  13.4 ± 7.7 \|14.0 ± 6.9  16.2 ± 8.0 \|3.01 ± 7.1  8.0 ± 7.7 \| 12.6 ± 7.1  3.3 ± 6.9 \| 5.5 ± 7.1  4.4 ± 7.4 \| 1.9 ± 7.1  **55 ± 15** \|29 ± 15  **7.7 ± 16.8** \|24 ± 15  **41.1 ± 15** \|28 ± 16  5.0 ± 14.9 \|9.1 ± 16  7.5 ± 14.9 \| 9.7 ± 15  8.6 ± 15.5 \| 8.0 ± 15  2.5 ± 1.5 \|5.3 ± 1.7  4.0 ± 2.0 \|2.5 ± 1.7  0.9 ± 1.6 \|2.7 ± 1.7  1.4 ± 1.6 \|1.8 ± 1.5  1.4 ± 1.5 \|0.7 ± 1.7  **12.6 ± 3.8** \|1.1 ± 3.7  **9.0 ± 4.9** \|1.3 ± 3.8  2.7 ± 3.8 \|5.5 ± 3.7  2.2 ± 3.3 \|2.5 ± 3.8  2.9 ± 3.7 \|2.8 ± 3.8  9.5 ± 4.0 \|3.5 ± 3.6  **12.1 ± 4.9**\|2.5 ± 3.7  2.8 ± 3.9 \|5.9 ± 3.7  2.9 ± 3.7 \|2.3 ± 3.8  4.0 ± 3.7 \|2.9 ± 3.7 |  |  |
| Smith and Kulig[37] | Turning while walking:   - Stance - Swing | Normalised amplitude | dMF  Lumbar Long  Thoracic Long  dMF  Lumbar Long  Thoracic Long | 1.3±0.1 (14)  1.3±0.1  1.0±0.3  0.5±0.3  0.5 ± 0.1  0.9±0.2 | 1.3±0.1 (14)  1.3±0.1  1.0±0.2  0.5±0.1  0.5 ± 0.2  1.0±0.3 | 0  0  0 (-0.74 to 0.74)  0 (-0.74 to 0.74)  0 (-0.74 to 0.74)  0.39 (-0.36 to 1.14) | **5** |
| Suehiro et al.[65] | Lifting task | ARV normalised (%MVC) | TrA/IO  Lumbar MF  EO  ES | 14.9 ± 7.4 (20)  27.1 ± 14.0  8.7 ± 8.9  26.7 ± 13.0 | 19.6 ± 10.7 (25)  32.7 ± 11.1  13.3 ± 18.0  37.5 ± 12.0 | 0.50 (-0.10 to 1.10)  0.45 (-0.15 to 1.04)  0.31 (-0.28 to 0.90)  **0.87 (0.25 to 1.48)^a^** | **2** |
| Sutherlin et al.[67] | ADM during   - Prone - Sitting - Standing - Walking   ADM during   - Supine - Sitting - Standing - Walking | Thickness Modulation  Thickness Modulation | Lumbar MF  TrA | 1.03 ± 0.06 (34)  1.02 ± 0.04  1.01 ± 0.04  1.01 ± 0.04  1.44 ± 0.24  1.28 ± 0.24  1.17 ± 0.24  1.32 ± 0.33 | 1.02 ± 0.05 (25)  1.01 ± 0.03  1.01 ± 0.04  1.01 ± 0.05  1.28 ± 0.23  1.12 ± 0.19  1.15 ± 0.19  1.25 ± 0.27 | -0.18 (-0.70 to 0.34)  -0.28 (-0.80 to 0.24)  0  0 (-0.52 to 0.52)  **-0.67 (-1.20 to -0.14)^a^**  **-0.73 (-1.26 to -0.19)^a^**  -0.09 (-0.61 to 0.43)  -0.23 (-0.75 to 0.29) | **4** |
| Viggiani et al.[33] | Trunk extension while standing | Commonality (RVC/RVC) | TES and LES bilat | 0.44±0.20(11) | 0.29±0.18(11) | **-0.78 (-1.64 to 0.09)^a^** | **2** |
| **Muscle activity** *- Timing* | | | | | | | |
| Hodges and Richardson[52] | Hip flexion  Hip extension | Latency Rectus femoris – Trunk muscles (ms)  Latency Gluteus Maximus – Trunk muscles (ms) | TrA  OI  OE  RA  ES  TrA  OI  OE  RA  ES | -86 ± 40 (15)  -48 ± 46  -14 ± 44  -27 ± 49  -69 ± 32  -71±35 (15)  -22±69  -20±79  -50±32  11±24 | 36 ± 55 (15)  -10 ± 46  -1 ± 46  7 ± 41  -23 ± 40  48±74 (15)  -14±63  4±62  -36±51  37±31 | **2.54 (1.58 to 3.50)^a^**  **0.83 (0.08 to 1.57)^a^**  0.29 (-0.43 to 1.01)  **0.75 (0.012 to 1.49)^a^**  **1.27 (0.49 to 2.05)^a^**  **2.06 (1.17 to 2.94)^a^**  0.12 (-0.60 to 0.84)  0.34 (-0.38 to 1.06)  0.33 (-0.39 to 1.05)  **0.94 (0.18 to 1.69)^a^** | **4**  *(continued on next page)* |
| Hodges and Richardson[51,54]      Hodges[53] | Rapid arm flexion  Rapid arm extension  Rapid arm abduction with neutral feedback | Latency Deltoid – Trunk muscles (ms)  Latency Deltoid – Trunk muscles (ms)  Latency Deltoid – Trunk muscles (ms) | TrA  OI  OE  RA  ES  TrA  OI  OE  RA  ES  TrA  OI  OE  RA | -39 ± 30 (15)  26 ± 52  57 ± 47  84 ± 59  9 ± 32  -25 ± 22 (15)  6 ± 33  30 ± 48  -10 ± 30  74 ± 54  -38.4 ± 32.5  18.1 ± 51.5  26.1 ± 65.1  58.7 ± 67.8 | 126 ± 104 (15)  80 ± 83  92 ± 42  124 ± 95  11 ± 31  54 ± 39 (15)  11 ± 23  41 ± 39  20 ± 18  70 ± 38  42.0 ± 37.9  25.4 ± 40.7  47.1 ± 35.2  79.7 ± 43.4 | **2.10 (1.21 to 3.07)^a^**  **0.79 (0.02 to 1.56)^a^**  **0.79 (0.04 to 1.53)^a^**  0.51 (-0.22 to 1.23)  0.07 (-0.64 to 0.79)  **2.50 (1.54 to 3.45)^a^**  0.18 (-0.54 to 0.89)  0.25 (-0.47 to 0.97)  **1.21 (0.43 to 1.99)^a^**  -0.09 (-0.80 to 0.63)  **2.28 (1.33 to 3.23)^a^**  0.16 (-0.58 to 0.90)  0.40 (-0.35 to 1.15)  0.37 (-0.38 to 1.12) | **4**  **4** |
| MacDonald et al.[13] | Rapid shoulder   - Flexion - Extension | Latency Deltoid – Trunk muscles (ms) | MF SF  MF LF  MF SF  MF LF | 2.09 ± 15.4 (19)  18.04 ± 15.7  55.72 ± 17.2  75.20 ± 16.8 | 21.10 ± 15.6 (15)  19.00 ± 15.3  90.34 ± 16.5  94.20 ± 15.9 | **1.23 (0.49 to 1.96)^a^**  0.06 (-0.62 to 0.74)  **2.05 (1.21 to 2.88)^a^**  **1.16 (0.43 to 1.89)^a^** | **2** |
| Smith and Kulig[37] | Turning while walking:   - Stance - Swing | Duration of activity  (% of turning phase) | dMF  Lumbar Longis  Thoracic Longis  dMF  Lumbar Longis  Thoracic Longis | 22.6±3.8 (14)  23.4±8.5  22.0±8.9  6.4±2.8  12.2±8.5  17.3±8.8 | 23.2±5.2 (14)  23.0±5.6  22.5±10  7.5±2.9  13.4±5.2  18.9±7.2 | 0.13 (-0.61 to 0.87)  -0.06 (-0.80 to 0.69)  0.05 (-0.69 to 0.79)  0.39 (-0.36 to 1.13)  0.17 (-0.57 to 0.91)  0.20 (-0.54 to 0.94) | **5** |
| Suehiro et al.[65] | Lifting task | Latency Deltoid – Trunk muscles (ms) | TrA/IO  Lumbar MF  EO  ES | 10.7 ± 19.9 (20)  −9.7 ± 12.2  67.8 ± 37.0  −1.2 ± 11.9 | 82.0 ± 50.8 (25)  11.0 ± 23.0  99.7 ± 66.5  11.0 ± 27.1 | **1.77 (1.08 to 2.47)^a^**  **1.09 (0.46 to 1.72)^a^**  0.57 (-0.02 to 1.18)  0.56 (-0.04 to 1.16) | **2** |
| **Spine kinematics -** *Range of motion* | | | | | | | |
| Crosbie et al.[43] | Reaching movements:  Cross-reach  Downward reach | Max displacement(°) in   - Extension - Flexion - Flexion   Max displacement(°) in   - Extension - Flexion - Flexion | Upper thoracic  Lower thoracic  Lumbar  Upper thoracic  Lower thoracic  Lumbar | 11.1 ± 7.1 (20)  16.4 ± 13.7  16.0 ± 8.0  2.8 ± 8.6  25.3 ± 12.8  22.9 ± 10.8 | 5.0 ± 5.6 (20)  8.9 ± 8.2  12.5 ± 11.4  0.7 ± 6.6  17.7 ± 12.3  17.9 ± 16.6 | **-0.95 (-1.61 to -0.30)^a^**  **-0.66 (-1.30 to -0.03)^a^**  -0.36 (-0.98 to 0.27)  *(continued on next page)*  -0.27 (-0.90 to 0.35)  -0.61 (-1.24 to 0.03)  -0.36 (-0.98 to 0.27) | **2** |
| Fenety and Kumar[47] | Standing:   - Full – flexion - Full – extension | Angle L1-S2 (°) | Lumbar spine | 61.2 ± 8.3 (12)  26.0 ± 9.0 | 55.4 ± 8.9 (10)  13.7 ± 4.7 | -0.68 (-1.54 to 0.19)  **-1.67 (-2.64 to -0.70)^a^**  *(continued on next page)* | **6** |
| Grimstone and Hodges[50] | Standing during quite breathing | Angle displacement (°) | Lumbopelvic region | 0.13±0.06 (10) | 0.24±0.28 (10) | 0.54 (-0.35 to 1.44) | **2** |
| Phillips[62] | Flex / Ext in sitting | Tot ROM (°) | Lumbar spine | 43.2 ± 13.6 (50) | 35.4 ± 10.9 (50) | **-0.63 (-1.03 to -0.23)^a^** | **2** |
| Viggiani et al.[33] | Trunk extension while standing | ROM in extension(°) | Trunk/pelvis angle | 21.8±9.7(11) | 23.1±6.3(11) | 0.16 (-0.68 to 1.00) | **2** |
| **Spine kinematics –** *Coordination & others* | | | | | | | |
| Smith and Kulig[64] | Turning while walking   - Stance - Swing | Angular deviation of the mean coupling angle (°) | Trunk and pelvis | 7.26 ± 1.95 (14)  22.20 ± 6.88 | 8.51 ± 2.42 (14)  24.89 ± 9.93 | 0.57 (-0.19 to 1.32)  0.31 (-0.43 to 1.06) | **5** |
| Sutherlin et al.[66] | Landing task | Change in joint moment to the change in angle | Lumbar spine | 0.10 (0.06, 0.34)^b^ (n=24) | 0.12 (0.05, 0.27)^b^  (n=21) | *p* = 0.86 | **4** |
| Crosbie et al.[43] | Reaching movements:  Cross-reach  Downward reach | Peak velocity (°/s) in:   - Extension - Flexion - Flexion   Peak velocity (°/s) in:   - Extension - Flexion - Flexion | Upper thoracic  Lower thoracic  Lumbar  Upper thoracic  Lower thoracic  Lumbar | 17.7 ± 8.9 (20)  26.1 ± 16.0  20.6 ± 7.8  3.9 ± 17.0  36.8 ± 21.9  28.2 ± 19.4 | 11.4 ± 5.8 (20)  16.7 ± 9.9  18.5 ± 12.3  0.4 ± 13.9  22.5 ± 22.3  23.3 ± 19.9 | **-0.84 (-1.49 to -0.19)^a^**  **-0.71 (-1.35 to -0.07)^a^**  -0.20 (-0.83 to 0.42)  -0.23 (-0.85 to 0.40)  **-0.65 (-1.23 to -0.01)^a^**  -0.25 (-0.87 to 0.37) | **2** |
| **Sensorimotor control –** *Proprioception* | | | | | | | |
| Elsig et al.[35] | Cervicocephalic relocation test | Joint position error (°) | Cervical spine | 2.67 ± 0.55 (30) | 3.25 ± 0.96 (30) | **0.58 (0.20 to 0.96)^a^** | **4** |
| Phillips[61] | Position awareness test:   - Standing - Sitting | Joint position error (°) | Lumbar spine  (L1-S1) | 0.86 ± 0.63 (40)  1.39 ± 0.56  *Log transform* | 0.68 ± 0.57 (61)  1.58 ± 0.65  *Log transform* | -0.30 (-0.70 to 0.10)  0.31 (-0.10 to 0.70)  *Log transform* | **3** |
| Phillips[62] | Position awareness test in sitting | Joint position error (°) | Lumbar spine  (L1-S1) | 3.48 ± 2.51 (50) | 3.95 ± 2.84 (50) | 0.18 (-0.22 to 0.57)  *(continued on next page)* | **2** |
| **Muscle properties -** *Tissue characteristics* | | | | | | | |
| D’Hooge et al.[45] | Rest, supine | Signal intensity:   - Total Muscle CSA - Lean muscle CSA - Muscle-fat index | MF  ES  PS  MF  ES  PS  MF  ES  PS | 37.5 ± 19.1 (13)  99.4 ± 16.2  73.0 ± 19.1  30.6 ± 17.5  89.5 ± 17.6  68.8 ± 19.0  14.0 ± 2.6  20.7 ± 2.5  21.9 ± 2.9 | 41.0 ± 15.7 (13)  96.1 ± 14.1  79.8 ± 17.6  34.6 ± 12.7  87.1 ± 15.1  75.3 ± 16.5  18.4 ± 6.4  23.9 ± 6.1  25.9 ± 5.9 | 0.20 (-0.57 to 0.97)  -0.22 (-0.99 to 0.55)  0.37 (-0.41 to 1.15)  0.26 (-0.51 to 1.03)  -0.15 (-0.92 to 0.62)  0.37 (-0.41 to 1.14)  -0.08 (-0.85 to 0.69)  -0.63 (-1.42 to 0.16)  0.17 (-0.60 to 0.94) | **2** |
| D’Hooge et al.[46] | Rest, supine  After exercise | Transverse relaxation times  (T2 rest)  Transverse relaxation times  (T2 shift) | MF  ES  QL  Psoas  MF  ES  QL  Psoas | - (13)  -  -  -  -  -  -  - | - (13)  -  -  -  -  -  -  - | **-2.08 (-4.09 to -0.06)^a^**  -0.04 (-2.39 to 2.30)  0.98 (-1.40 to 3.35)  -0.71 (-2.14 to 0.71)  **1.31 (0.11 to 2.51)^a^**  0.57 (-0.74 to 1.87)  -0.57 (-2.14 to 1.00)  0.75 (-0.14 to 1.65 | **2** |
| Nagar et al.[58] | Quiet standing | Muscle thickness (mm) | TrA | 4.52±1.78 (18) | 6.03±1.31 (18) | **0.97 (0.28 to 1.66)^a^** | **3** |
| **Neuromuscular performance -** *Strength & Endurance* | | | | | | | |
| Applegate et al.[40,41] | Sorensen test  Trunk extension | Time to task failure (s)  MPF slope (%/sec)  Strength – MVC (N)  Trunk moment(N∙m) | Back extensors  Iliocostalis Lumb  Back extensors  Back extensors | 120.9 ± 52.9 (24)  −0.4 ± 0.01  476.7 ± 258.7  42.3 ± 20.1 | 116.3 ± 43.1 (24)  −0.4 ± 0.01  511.7 ± 173.4  45.4 ± 26.5 | -0.095 (-0.66 to 0.47)  0  0.16 (-0.41 to 0.73)  0.13 (-0.43 to 0.70) | **4** |
| D’Hooge et al.[46] | Low-load trunk extension exercise | Borg scale (6-20) | Back extensor | 9.4±1.6 (13) | 10.8±1.0 (13) | **1.05 (0.23 to 1.87)^a^** | **2** |
| Elsig et al.[35] | Craniocervical Flexion Test | Pressure level (mmHg) | Deep neck flexors | 28.07 ± 2.85 (30) | 25.87 ± 2.73 (30) | **-2.2 (-3.6 to -0.8)^a^** | **4** |
| Janssens et al.[39] | Magnetic phrenic nerve stimulation   - Before exercise - After exercise (inspiratory muscle loading) | Potentiated twitch transdiaphragmatic pressures (cm H2O)  Baseline decrease  >10 % (of the twitch transdiaphragmatic pressure) | Diaphragm | 44±7 (10)  N = 8/10 | 51 ± 14 (10)  N = 4/10 | 0.63 (-0.27 to 1.53)  *(continued on next page)*  RR=2.0 (0.88 to 4.54) | **2** |
| Johanson et al.[55] | Sorensen test | Time to task failure (s)  Borg scale (0-10)  MPF (end) | Back extensors  Lumbar MF | 184.0±40.4 s (16)  8.3±1.3  44.9 ± 13.4 | 127.4±36.7 s (16)  9.2±1.2  55.7 ± 14.4 | **-1.47 (-2.25 to -0.69)^a^**  0.73 (-0.02 to 1.43)  **0.78 (0.058 to 1.50)^a^** | **3** |
| Fenety and Kumar[47] | Isokinetic trunk   - Flex (concentric) - Flex (eccentric) - Ext (concentric) - Ext (eccentric) | Average torque (Nm) | Trunk flexors  Trunk extensors | 1.55 ± 0.24 (12)  1.85 ± 0.24  2.43 ± 0.42  3.23 ± 0.45 | 1.63 ± 0.16 (10)  1.84 ± 0.32  2.41 ± 0.38  2.78 ± 0.63 | 0.38 (-0.46 to 1.23)  -0.04 (-0.88 to 0.80)  -0.05 (-0.89 to 0.79)  -0.84 (-1.71 to 0.04) | **6** |
|  | | | | | | | |

Abbreviations: ADM, abdominal drawing-in manoeuvre; ASLR, active straight leg raise; CLR, crook-lying active leg raise; dMF, deep multifidus fibres; ES, erector spinae; MF, multifidus; MFlf, multifidus long fibres; MFsf, multifidus short fibres; MPF, mean power frequency; NS, not significative; OE, external oblique; OI, internal oblique; PM-t, psoas major transverse process; PM-v, psoas major vertebral body; PSLR, prone straight leg raise; QL-a, quadratus lumborum anterior; QL-p, quadratus lumborum posterior; RA, rectus abdominis; RMS, root mean square; TrA, transversus abdominis.

^a^ Statistically significant

^b^ Value reported as median and interquartile range
