# Supplementary material for: Are neuromuscular adaptations present in people with recurrent spinal pain during a period of remission? a systematic review
Source: PLoS One. 2021 Apr 1;16(4):e0249220. doi: 10.1371/journal.pone.0249220 (PMC8016280; doi:10.1371/journal.pone.0249220)
Supplement: S1 File — (DOCX) [file pone.0249220.s004.docx]

**S1 File. Search strategy used in MEDLINE (OVID interface)**

1. (recurren* or history or episod* or intermittent or fluctuat* or relaps* or flare).mp.

2. Back Pain/ or (back pain or back ache* or backache*).mp.

3. exp Low Back Pain/ or (low* back pain or lumbago or LBP or lumbar pain or lumbar spine pain).mp.

4. (dorsal pain or thoracic pain).mp.

5. Neck Pain/ or (neck pain* or neckache* or neck ache* or cervical pain* or cervicalgia*).mp.

6. (spin* pain or spin* injur*).mp.

7. exp neck injuries/ or (neck injur* or cervical injur* or whiplash or WAD).mp.

8. back injuries/ or back injur*.mp.

9. 2 or 3 or 4 or 5 or 6 or 7 or 8

10. 1 and 9

11. exp Range of motion, Articular/

12. (flexibility or range of motion or mobility or ROM or AROM or PROM).mp.

13. (flexion or extension or lateral flexion or sidebending or rotation).mp.

14. (inclinometer or inclinometry or goniometer).mp.

15. exp biomechanical phenomena/

16. (biomechanic* or kinematic*).mp.

17. timing.mp.

18. (opto-electronic or camera* or inertial or accelerom* or movement analysis or motion analysis or IMU*).mp.

19. exp proprioception/

20. Somatosensory disorders/

21. (position adj3 sense*).mp.

22. (propriocept* or kin?esthe* or Joint position* or Joint reposition* or Reposition*).mp.

23. (Somatosensory disorder* or Sensorimotor or Sensory motor or Somatosensory control).mp.

24. Sensor* feedback.mp.

25. exp psychomotor performance/

26. Movement/

27. Mot* activit*.mp.

28. Coordination*.mp.

29. (Movement* Accurac* or Motion* Accurac* or Movement* Variabilit* or Motion* Variabilit* or Steadiness or Smoothness or Motion* precision* or Movement* precision*).mp.

30. Motor skill*.mp.

31. 11 or 12 or … 29 or 30

32. (musc* activit* or neuromusc* activit*).mp.

33. (musc* control* or neuromusc* control*).mp.

34. perturbation*.mp.

35. (rapid movement* or rapid motion*).mp.

36. (musc* adj3 tim*).mp.

37. musc* contraction*.mp.

38. exp Electromyography/

39. (Electromyo* or EMG or sEMG or surface EMG or bipolar EMG).mp.

40. (intramuscular EMG or iEMG or fine wire or fine-wire).mp.

41. (high density EMG or HDEMG or HD-EMG or HDsEMG or HD-sEMG).mp.

42. 32 or 33 … or 40 or 41

43. exp Muscle contraction/

44. exp muscle strength/

45. muscle fatigue/

46. exp physical endurance/

47. physical exertion/

48. Physical fitness/

49. (Strength* or Endurance or Fatigue or Force*).mp.

50. muscle strength dynamometer/

51. dynamomet*.mp.

52. 43 or 44 or 45 or 46 or 47 or 48 or 49 or 50 or 51

53. exp muscle fibers, skeletal/

54. exp adipose tissue/

55. connective tissue/

56. musc* atroph*.mp.

57. (cross-sectional area or cross sectional area or CSA or musc* size or musc* thickness).mp.

58. (adipose tissue or fat* infiltration or fat* deposition or intramuscular fat or fat* tissue or musc* fibre*).mp.

59. (musc* fiber* or fiber type* or musc* morpholog* or musc* composition or musc* structur*).mp.

60. 53 or 54 or 55 or 56 or 57 or 58 or 59

61. 31 or 42 or 52 or 60

62. 10 and 61
