# Supplementary material for: Are neuromuscular adaptations present in people with recurrent spinal pain during a period of remission? a systematic review
Source: PLoS One. 2021 Apr 1;16(4):e0249220. doi: 10.1371/journal.pone.0249220 (PMC8016280; doi:10.1371/journal.pone.0249220)
Supplement: S2 File — (DOCX) [file pone.0249220.s005.docx]

| **S2 File. Contacted authors and studies** | | | |
| --- | --- | --- | --- |
| **AUTHOR CONTACTED** | **STUDIES** | **REPLY (YES/NO)** |  |
| Applagate, Megan E | - Sørensen test performance is driven by different physiological and psychological variables in participants with and without recurrent low back pain. J Electromyogr Kinesiol. 2019 Feb;44:1-7. doi: 10.1016/j.jelekin.2018.11.006. Epub 2018 Nov 9. PMID: 30447543. | **Y** |  |
| Elsig, Simone | - Sensorimotor tests, such as movement control and laterality judgment accuracy, in persons with recurrent neck pain and controls. A case-control study. Man Ther. 2014 Dec;19(6):555-61. doi: 10.1016/j.math.2014.05.014. | **Y** |  |
| He, Jade | - Characterizing Lumbar Multifidus Fatty Infiltration with MRI: Is there a correct Region of Interest? Journal of Orthopaedic Research. Conference. 2016;34 | **N** |  |
| Hides, Julie A | - A magnetic resonance imaging investigation of the transversus abdominis muscle during drawing-in of the abdominal wall in elite Australian Football League players with and without low back pain. J Orthop Sports Phys Ther. 2010;40(1):4–10. | **N** |  |
| Hodges, Paul | - Changes in recruitment of the abdominal muscles in people with low back pain: ultrasound measurement of muscle activity. Spine (Phila Pa 1976). 2004 Nov 15;29(22):2560-6. doi: 10.1097/01.brs.0000144410.89182.f9. | **Y** |  |
| Janssens, Lotte | - Greater diaphragm fatigability in individuals with recurrent low back pain. Respir Physiol Neurobiol. 2013 Aug 15;188(2):119-23. doi: 10.1016/j.resp.2013.05.028. Epub 2013 May 31. PMID: 23727158 | **Y** |  |
| Lee, JH | - Measurement of muscle cross-sectional area of the trunk and the lower extremities in subjects with history of low back pain. JNMS. 1996;4(4):131-136 | **N** |  |
| McCreesh, Karen | - A study to determine whether rowers with a history of low back pain shows a reduced endurance time of transversus abdominus compared to rowers without such a history. Physiotherapy Ireland. 2007;28(1):48 | **Y** |  |
| Sung, Paul S | - Analysis of relative kinematic index with normalized standing time between subjects with and without recurrent low back pain. Eur Spine J. 2017;26(2):518–527 - Trunk Reaction Time and Kinematic Changes Following Slip Perturbations in Subjects with Recurrent Low Back Pain [published correction appears in Ann Biomed Eng. 2019 Jan;47(1):333]. Ann Biomed Eng. 2018;46(3):488–497. - Trunk sway response to consecutive slip perturbations between subjects with and without recurrent low back pain. Musculoskelet Sci Pract. 2018;33:84–89. - Different parts of erector spinae muscle fatigability in subjects with and without low back pain. Spine J. 2009;9(2):115–120. - Kinematic chain reactions on trunk and dynamic postural steadiness in subjects with recurrent low back pain. J Biomech. 2017;59:109–115. | **N** |  |
| Viggiani, Daniel | - A comparison of trunk control in people with no history, standing-induced, and recurrent low back pain during trunk extension. J Man Manip Ther. 2020 May;28(2):94-102. | **Y** |  |
