# Supplementary material for: Are neuromuscular adaptations present in people with recurrent spinal pain during a period of remission? a systematic review
Source: PLoS One. 2021 Apr 1;16(4):e0249220. doi: 10.1371/journal.pone.0249220 (PMC8016280; doi:10.1371/journal.pone.0249220)
Supplement: S3 File — (DOCX) [file pone.0249220.s006.docx]

**S3 File. Collated records**

- Applegate ME, France CR, Russ DW, Leitkam ST, Thomas JS. Sørensen test performance is driven by different physiological and psychological variables in participants with and without recurrent low back pain. J Electromyogr Kinesiol [Internet]. 2019;44:1–7. Available from: https://doi.org/10.1016/j.jelekin.2018.11.006
- Applegate M, France C, Russ D, Leitkam S, Thomas J. Classic and modified sorensen test performance is driven by different physiological and psychological variables in participants with and without recurrent low back pain. Pain Pract. 2018;18(1):86
- Applegate ME, France CR, Russ DW, Leitkam ST, Thomas S. Determining Physiological and Psychological Predictors of Time to Task Failure on a Virtual Reality Sørensen Test in Participants With and Without Recurrent Low Back Pain : Exploratory Study. JMIR Serious Games. 2018;6(3):e10522
- D’hooge R, Cagnie B, Crombez G, Vanderstraeten G, Dolphens M DL. Increased intramuscular fatty in fi ltration without differences in lumbar muscle cross-sectional area during remission of unilateral recurrent low back pain. Man Ther [Internet]. 2012;17(6):584–8. Available from: http://dx.doi.org/10.1016/j.math.2012.06.007
- D’Hooge R, Cagnie B, Crombez G, Vanderstraeten G, Achten E, Danneels L. Lumbar muscle dysfunction during remission of unilateral recurrent nonspecific low-back pain: Evaluation with muscle functional MRI. Clin J Pain. 2013;29(3):187–94
- D'Hooge R, Cagnie B, Danneels L. Persistent lumbar muscle dysfunction in remission of recurrent low back pain-evaluation with muscle functional MRI. Physiotherapy. 2011;97(SUPPL. 1),p.eS260-es261
- Hodges PW RC. Inefficient muscular stabilization of the lumbar spine associated with low back pain. A motor control evaluation of transversus abdominis. Spine (Phila Pa 1976). 1996;21(22):2640‐2650.
- Hodges PW RC. Altered trunk muscle recruitment in people with low back pain with upper limb movement at different speeds. Arch Phys Med Rehabil. 1999;80(9):1005–12
- Hodges PW RC. Delayed postural contraction of transversus abdominis in LBP associated with mov of the lower limb. J Spinal Disord. 1998;11(1):46‐56
- Hodges PW. Changes in motor planning of feedforward postural responses of the trunk muscles in low back pain. Exp Brain Res. 2001;141(2):261–6.
- Smith JA, Kulig K. Altered Multifidus Recruitment During Walking in Young Asymptomatic Individuals With a History of Low Back Pain. J Orthop Sport Phys Ther. 2016;46(5):365–74
- Smith JA, Kulig K. Trunk-pelvis coordination during turning: A cross sectional study of young adults with and without a history of low back pain. Clin Biomech. 2016;36:58–64
- Smith JA, Gordon J KK. The influence of divided attention on walking turns: Effects on gait control in young adults with and without a history of low back pain. Gait Posture. 2017;58:498–503
- Park RJ, Tsao H, Claus A, Cresswell AG, Hodges PW. Recruitment of Discrete Regions of the Psoas Major and Quadratus Lumborum Muscles Is Changed in Specific Sitting Postures in Individuals With Recurrent Low Back Pain. J Orthop Sport Phys Ther [Internet]. 2013;43(11):833–40. Available from: http://www.jospt.org/doi/10.2519/jospt.2013.4840
- Park RJ, Tsao H, Cresswell AG, Hodges PW. Changes in direction-specific activity of psoas major and quadratus lumborum in people with recurring back pain differ between muscle regions and patient groups. J Electromyogr Kinesiol [Internet]. 2013;23(3):734–40. Available from: http://dx.doi.org/10.1016/j.jelekin.2013.01.010
